# Contrasting mechanisms of growth in two model rod-shaped bacteria

**DOI:** 10.1038/ncomms15370

**Published:** 2017-06-07

**Authors:** Cyrille Billaudeau, Arnaud Chastanet, Zhizhong Yao, Charlène Cornilleau, Nicolas Mirouze, Vincent Fromion, Rut Carballido-López

**Affiliations:** 1 Micalis Institute, INRA, AgroParisTech, Université Paris-Saclay, 78350 Jouy-en-Josas, France; 2 MaIAGE, INRA, Université Paris-Saclay, Jouy-en-Josas F78350, France

## Abstract

How cells control their shape and size is a long-standing question in cell biology. Many rod-shaped bacteria elongate their sidewalls by the action of cell wall synthesizing machineries that are associated to actin-like MreB cortical patches. However, little is known about how elongation is regulated to enable varied growth rates and sizes. Here we use total internal reflection fluorescence microscopy and single-particle tracking to visualize MreB isoforms, as a proxy for cell wall synthesis, in *Bacillus subtilis* and *Escherichia coli* cells growing in different media and during nutrient upshift. We find that these two model organisms appear to use orthogonal strategies to adapt to growth regime variations: *B. subtilis* regulates MreB patch speed, while *E. coli* may mainly regulate the production capacity of MreB-associated cell wall machineries. We present numerical models that link MreB-mediated sidewall synthesis and cell elongation, and argue that the distinct regulatory mechanism employed might reflect the different cell wall integrity constraints in Gram-positive and Gram-negative bacteria.

## Abstract (web summary)

Protein MreB participates in elongation of sidewalls during growth of most rod-shaped bacteria. Here, the authors use fluorescence microscopy and single-particle tracking to visualize MreB, showing that *Bacillus subtilis* and *Escherichia coli* appear to use different strategies to adapt to growth rate variations.

How cell size and growth rate are regulated is a fundamental but poorly understood question in cell biology, for both eukaryotic and prokaryotic systems. Pioneering studies in model microbial organisms conducted by the ‘Copenhagen school' measured the changes in macromolecular composition of the cell (DNA, RNA, proteins and biomass), depending on different carbon sources or supplements in the growth media. It was shown that bacterial cells of identical genetic background redefine steady-state average cell size and chemical composition in response to changing growth rate^1^,^2^. For example, cells of the rod-shaped Gram-positive model bacterium *Bacillus subtilis* grow up to six times as fast and twice as long when grown in rich medium compared to poor medium^3^. Because most of these studies were performed at the infancy of molecular and cell biology, the molecular mechanisms by which each cellular component is coordinated with cell growth and division were not addressed. Furthermore, experiments were carried out by bulk measurements of cell populations^1^,^2^,^3^,^4^,^5^,^6^,^7^; therefore, cell size and growth rate regulation at the single-cell level remained an outstanding question.

As a hallmark of microbial life, the peptidoglycan (PG) sacculus is the most conspicuous macromolecule expanding in concert with cell growth. This three-dimensional biopolymer mesh, composed of linear glycan chains cross-linked by peptide bridges^8^, provides physical integrity by balancing turgor pressure and maintains cell morphology. Although the chemical composition of PG is highly conserved in almost all bacteria, Gram-positive bacteria have substantially thicker cell walls (responsible for retaining the Gram stain) than their Gram-negative counterparts (20–35 nm for *B. subtilis* and 2–7 nm for the rod-shaped model Gram-negative bacterium *Escherichia coli*)^9^,^10^,^11^. It was therefore postulated that *E. coli* has a planar monolayer of PG, whereas *B. subtilis* has multiple concentric layers of PG^12^. In response to nutrient-imposed changes in growth rate, both *B. subtilis* and *E. coli* modulate their average length^4^,^13^. However, while *B. subtilis* cells maintain a remarkably constant diameter in all growth conditions (‘constrained hoop' model)^4^,^14^, *E. coli* cells adjust their average length as well as width to keep a roughly constant length-to-width ratio when accommodating the change in mass (‘constant shape' model)^6^,^15^.

Despite these differences, decades of research have revealed a similar mechanism of PG synthesis in the evolutionarily distant *B. subtilis* and *E. coli*. Cell cycle progression starts by elongation of the cylindrical sidewall and ends with division by formation of a crosswall (septum) at midcell^16^. According to the so-called ‘two-competing-sites model'^17^,^18^, there are two spatially specific and mutually exclusive pathways for PG synthesis: one for elongation (sidewall-specific) and one for division (septum-specific). Dedicated PG-synthesizing machineries, associated with different cytoskeletal elements that assemble beneath the cytoplasmic membrane, carry out septum and sidewall synthesis in a zonal and a diffusive manner, respectively. The tubulin homologue FtsZ assembles into a cytokinetic ring (the Z ring) at the future sites of division, and sequentially recruits other components of the septal PG machinery^19^,^20^. The PG elongation machinery (PGEM), on the other hand, is controlled by the actin homologue MreB^21^,^22^. MreB is widely distributed in bacteria with non-coccoid shapes^23^,^24^,^25^, and multiple *mreB* paralogues are often present in the genome of Gram-positive organisms. For example, *B. subtilis* has three MreB isoforms, namely MreB, Mbl (MreB-like) and MreBH (MreB-homologue).

*In vitro*, MreB proteins self-assemble into filaments that bind directly to membranes^26^,^27^,^28^. Early subcellular localization studies suggested that MreB forms long filamentous structures running the length of the sidewalls along a helical path^23^,^29^. Similar to the role that the FtsZ ring plays in coordinating the divisome, MreB helices were thought to have a structural role in the spatial control of the PGEM^30^. However, recent higher-resolution light microscopy studies showed that in exponentially growing cells MreB proteins do not form such long-range helical structures^31^,^32^,^33^. Instead, they form disconnected assemblies that move processively around the cell diameter, along tracks compatible with the incorporation pattern of PG precursors into the sidewall^25^,^31^. It was also shown that MreB co-localizes and moves together with other components of the PGEM^31^,^34^, including the essential PG transglycosylase RodA^35^, which provides polymerization activity, and the PG transpeptidases PbpH and PBP2A^36^, which provide crosslinking activity. Furthermore, MreB motion is dependent on PG synthesis^31^,^32^, indicating that it parallels the action of the PGEM and thus reflects sidewall synthesis. Although the ultrastructure of MreB assemblies *in vivo* remains controversial, the current model proposes that they are membrane-associated scaffolds that spatially coordinate extra- and intra-cellular PG-synthesizing enzymes to ensure controlled cylindrical expansion of the sacculus^31^,^32^,^37^,^38^. However, it is still unclear how these presumed individual PGEMs surmount the formidable task of weaving an intact mesh network three orders of magnitude larger than themselves. It is also unknown how PG synthesis is regulated to enable different growth rates and cell sizes. Yet a cell that is growing six times faster and is twofold larger must have a twelvefold increase in the rate of PG synthesis. Lastly, it remains to be elucidated how a similar mechanism of sidewall elongation can give rise to distinct cell wall structures in Gram-positive and Gram-negative bacteria.

There are various strategies that cells could potentially adopt to regulate PGEM synthetic activity to achieve different growth rates and cell sizes: to change the number of active machines per unit area, to change their unit production speed or their unit production capacity (amount of PG inserted per machine), or a mix of those. Previous studies primarily focused on the analysis of MreB circumferential motion, largely ignoring the whole population of MreB assemblies and the fraction of these potentially exhibiting other dynamic behaviours.

Here we use time-lapse total internal reflection fluorescence microscopy (TIRFM) combined with single-particle tracking (SPT) analysis to characterize the localization and dynamics of membrane-associated MreB in *B. subtilis* and *E. coli* cells growing in different media as well as during nutrient upshift. Interestingly, only a fraction of MreB patches exhibits canonical circumferential movement in all conditions examined. Taking this fraction as a proxy for PG synthesis, we then look for correlation of patch speed and density with cell dimensions and growth rate. Our results indicate that these two model rod-shaped bacteria appear to employ distinct strategies in response to nutrient availability: *B. subtilis* may regulate speed of MreB patches, while *E. coli* may primarily regulate the amount of PG inserted per MreB patch.

## Results

### MreB forms patches close to the diffraction limit by TIRFM

To observe native adaptation of the localization and dynamics of MreB assemblies to different growth rates, we monitored MreB-fluorescent protein fusions expressed at the native loci under native regulation, as the only copy of the corresponding *mreB* in the genome, in nutrient-rich (LB) and nutrient-poor (S) media at 37 °C. For *B. subtilis*, we focused on the essential *mreB* and *mbl* genes, which are highly expressed during exponential growth^23^,^39^. The third paralogue, *mreBH*, is virtually not expressed and does not have a major role in cell morphogenesis under normal growth conditions^39^,^40^. These expression profiles were confirmed in our experimental conditions (Supplementary Fig. 1). For *E. coli*, we used a monomeric-superfolder-green fluorescent protein internal ‘sandwich' fusion (MreB-msfGFP^SW^) considered to be the most functional fluorescent protein fusion to MreB in this organism^41^ and used to analyse MreB dynamics in the most recent studies^42^,^43^,^44^. Viability, growth rate and cell morphology of the strains bearing these fluorescent fusions were indistinguishable from their respective wild-type strains in both media (Supplementary Fig. 2 and Supplementary Tables 1 and 2), indicating that the fusions are functional. Cell dimensions (average cell width and length) were only slightly affected (Supplementary Tables 1 and 2). The three MreB isoforms of *B. subtilis* have been shown to have partially redundant roles and overexpression of any one of them can, independently, support cell viability and rod-shape morphology in the absence of the other two^45^. It was therefore possible that our green fluorescent protein (GFP) fusions were only partially functional and that one or the other two isoforms were upregulated to compensate. Western blot analysis showed that, while *mreBH* was indeed highly upregulated in Δ*mreB* mutant cells (Supplementary Fig. 3a), in the strains bearing the native GFP fusions neither of the other two isoforms was compensating (Supplementary Fig. 3a–c). We concluded that the three fusions are minimally perturbative in the conditions used in our study.

**Figure 1:**
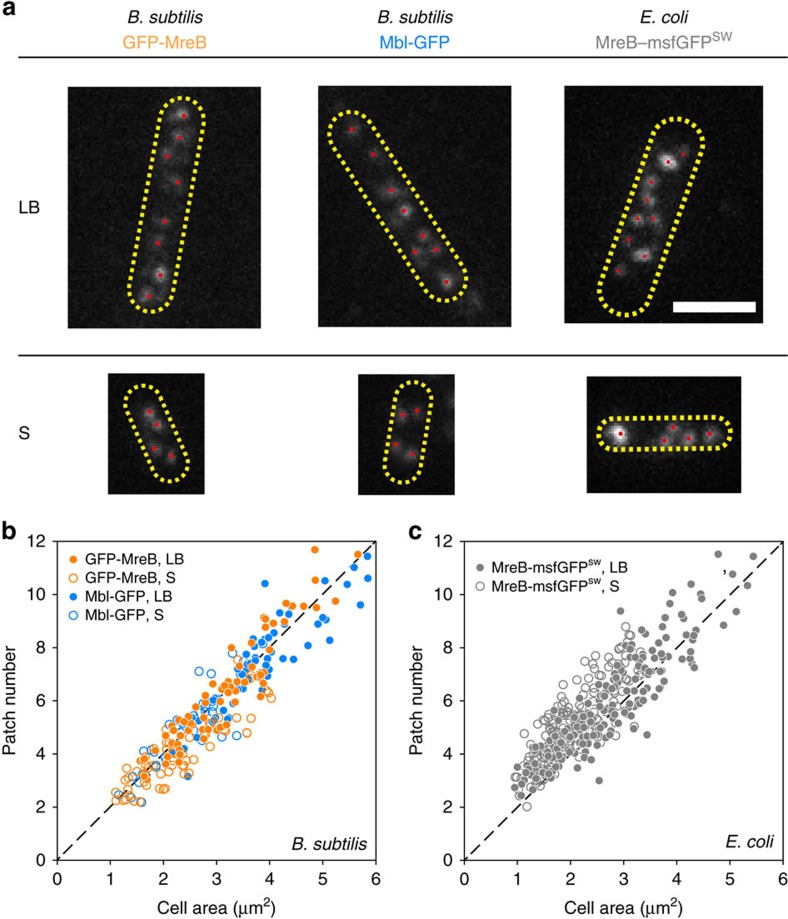
*B. subtilis* and *>E. coli>* >maintain constant patch density regardless of the nutrient conditions. (**a**) Patch identification on TIRFM images of representative *B. subtilis* cells natively expressing GFP-MreB and Mbl-GFP, and *E. coli* cells expressing MreB-msfGFP^SW^ grown in LB and S media. Cell contours were drawn based on the MIP of the TIRFM section. Scale bar, 2 μm. (**b**,**c**) Correlation of average patch number versus cell area in *B. subtilis* (**b**) and *E. coli* (**c**) cells. Dashed line, *y*=2*x*. Data are a compilation of at least two independent experiments.

We then used TIRFM, a sensitive method for studying molecular events at cell surfaces with high temporal resolution^46^, to characterize the localization and dynamics of membrane-associated MreB assemblies. In both *B. subtilis* and *E. coli*, MreB proteins assembled into spherical or elliptical patches close to the diffraction limit (TIRFM lateral resolution ∼250–300 nm) that were well separated along the cell cylinder (Fig. 1a). It is important to note that our wording ‘patch' does not exclude a filamentous ultrastructure for MreB assemblies. It only reflects their appearance when visualized by conventional light microscopy. MreB filaments shorter than 250–300 nm (that is, diffraction-limited) also appear as the spherical or elliptical patches reported here, regardless of their length^47^.

### MreB patch density is constant regardless of the growth rate

To quantify the number of MreB patches at the single-cell level, we accurately identified the centroids of all patches in the TIRFM section of each frame from time-lapse movies of individual cells (see Methods section, Supplementary Methods and Supplementary Figs 4 and 5a–c). In parallel, we measured the two-dimensional (2D) cell surface area under TIRF illumination by integrating the number of pixels within the cell contour obtained from the maximum intensity projection (MIP) of the corresponding movie (Supplementary Fig. 4d). Linear correlation between cell area and average patch number detected under TIRF illumination indicated that both *B. subtilis* and *E. coli* cells maintain a constant MreB/Mbl patch density (*ρ*) of ∼2 patches μm^−2^ (Fig. 1b,c), despite a twofold difference in growth rate between the two media (Supplementary Table 2). Comparable patch density was obtained in *B. subtilis* when inducible GFP fusions overexpressed relative to our native fusions (Supplementary Fig. 3d) were used (Supplementary Fig. 6a), indicating that the density of MreB patches on the cell surface is not affected by protein levels. Based on the average cell length and cell width in each growth medium (Supplementary Table 2), the total number of patches per cell can be extrapolated giving a minimum value of ∼10 patches per cell in S, and ∼21 patches per cell in LB. This is consistent with the assumed TIRFM penetration depth of ∼200 nm (ref. 46), illuminating roughly the bottom third of the bacterial cell in our microscopy set-up. These are to be considered relative numbers as some small MreB assemblies might be masked by noise in our imaging set-up. Also, we cannot exclude that multiple independent MreB assemblies are present in the patches when these are diffraction-limited.

### Only a fraction of all patches exhibits circumferential motion

**Figure 2:**
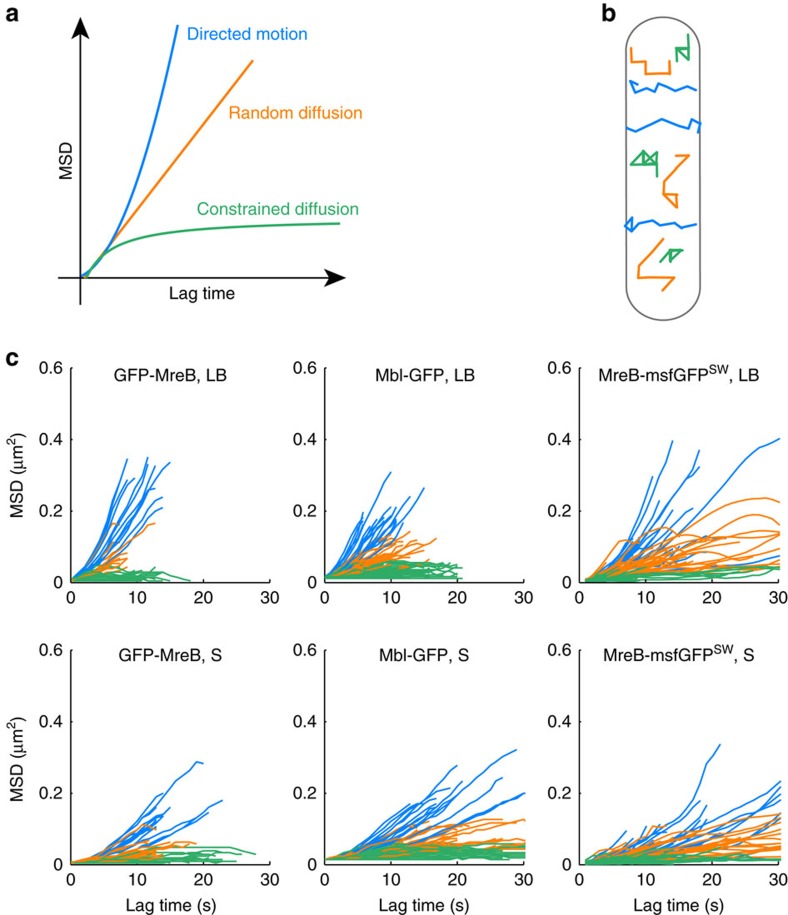
Heterogeneity of modes of movement of MreB patches at the single-cell level. (**a**,**b**) Idealized representation of the three categories of MreB patch motion. (**a**) MSD curves allow classification of the mode of movement: (i) second-order polynomial fit indicates directed motion (blue): MSD(*t*)=(*νt*)^2^, where *ν* is speed and *t* is time; (ii) a linear plot indicates random diffusion (orange): MSD(*t*)=4*Dt*, where *D* is the diffusion constant; (iii) when the MSD curve reaches a plateau, the system is undergoing constrained diffusion (green). (**b**) Representation of 2D-SPT trajectories (following the above-mentioned colour code) exhibiting the three modes of MreB patch motion in a rod-shaped cell. (**c**) Dynamics of MreB patches in individual cells growing at different growth rates. Samples of MSD curves of representative *B. subtilis* cells carrying native GFP-MreB (left) and Mbl-GFP (middle) fusions, and of *E. coli* cells carrying the MreB-msfGFP^SW^ fusion (right) grown in LB and S media at 37 °C. Blue, directed motion; orange, random diffusion; green, constrained diffusion.

Previous live-cell imaging studies on MreB dynamics focused on circumferentially mobile patches. MreB motion was analysed from kymographs drawn either across the cell width^32^ or along the tracks visible in maximum projections^31^, or by SPT of linear cell diameter spanning trajectories^32^,^33^,^42^. Thus, only patches exhibiting directed-movement across the cell were selected (Supplementary Fig. 7a, top panels). Non-mobile patches (Supplementary Fig. 7a, bottom) and/or patches potentially exhibiting other dynamic behaviours were excluded from further analysis. To enable a more systematic and unbiased study, we used automated 2D-SPT analysis to characterize and quantify the motion of all MreB patches in the TIRFM sections. This analysis confirmed that growing cells display subpopulations of both mobile and non-mobile MreB patches (Supplementary Fig. 7b). Next, we applied mean squared displacement (MSD) analysis to classify the types of MreB dynamic behaviour (see Supplementary Methods and Supplementary Fig. 8). MSD analysis of single trajectories typically allows to determine whether a particle is (1) moving with a dedicated trajectory (directed motion, which displays a quadratic curve in MSD versus *t* plots); (2) moving randomly due to Brownian motion (random diffusion, which displays a linear MSD feature); and (3) trapped and/or with limited movement, not freely diffusing (constrained diffusion, the MSD saturates and has a concave curvature) (Fig. 2a,b). The three types of motion were found among MreB traces for both *B. subtilis* and *E. coli* cells growing in either LB or S (>300 trajectories in time lapses of >25 cells for each fusion and growth medium, from at least three independent experiments per condition) (Fig. 2c and Supplementary Fig. 9a–c). This heterogeneity in the mode of movement supports the model where MreB assemblies function as disconnected entities, and not as long-range filament structures as currently debated in the field^22^,^30^. For a small fraction of traces (<15%, Supplementary Table 3), the behaviour was not sufficiently distinct to unambiguously assign a dynamic mode. This might be due to limitations of our experimental set-up, bad fitting and/or to mixed patterns of motions (for example, Supplementary Fig. 9d). Such trajectories were assigned to the unclassified category and discarded from further analysis. Dynamic behaviours of MreB patches, such as crossover, pause and reversal, were previously reported^31^,^32^,^37^. However, careful examination of 312 kymograph traces showed very few pauses (<2%) and arguably one potential reversal. Crossings of rotating patches, recently reported to be exceedingly rare events too^37^, represented 7% of the traces in our analysis. We concluded that such behaviours are infrequent and their effect on our statistical analysis negligible.

**Figure 3:**
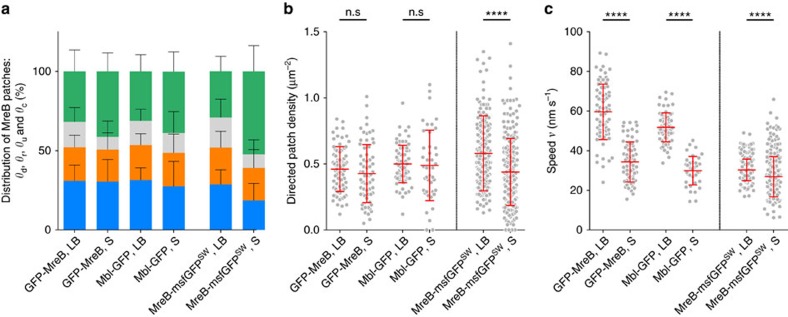
*B. subtilis* reduces the speed of directed patches in response to poor media, whereas *E. coli* mainly reduces their density. Quantification of MreB patch subpopulations and dynamics in *B. subtilis* (GFP-MreB and Mbl-GFP) and *E. coli* (MreB-msfGFP^SW^) cells exponentially growing in LB (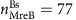, 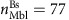 and 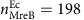) and S (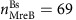, 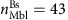 and 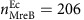) media. (**a**) Percentage of MreB patches per cell displaying directed motion (*θ*_d_, blue), random diffusion (*θ*_r_, orange), constrained diffusion (*θ*_c_, green) and unclassified motion (*θ*_u_, grey). Error bars correspond to the s.d. (**b**,**c**) Density (**b**) and speed distributions (**c**) of directed MreB patches. Data are a compilation of at least two independent experiments. Averages are shown in red; error bars correspond to the s.d. Statistical significance between LB and S media was calculated using the Mann–Whitney non parametric test (****=*P*<0.0001; ***=0.0001<*P*<0.001; **=0.001<*P*<0.01; *=0.01<*P*<0.05; ns=*P*>0.05).

Remarkably, in *B. subtilis* the fraction of MreB assemblies exhibiting directed motion (*θ*_d_), which corresponds to the canonical circumferential movement described previously^31^,^32^,^33^, was constant regardless of the fusion and growth medium (Fig. 3a,b and Supplementary Table 3). However, in *E. coli* a mild but statistically significant decrease of *θ*_d_ and thus of MreB-directed patch density was observed in slowly growing cells relative to rapidly growing cells (Fig. 3a,b and Supplementary Table 3). With the detection and reconnection parameters used in our SPT analysis, *θ*_d_ corresponded to approximately one-third of all MreB patches in the cell in *B. subtilis* (all media) and in *E. coli* cells growing in LB (Fig. 3a, see also Supplementary Table 3). These numbers are consistent with a recent analysis of MreB dynamics in *E. coli* cells growing in rich medium by single-molecule tracking, where the number of MreB tracks classified as having directed motion was found to be about 30% of the total number of tracks detected^42^. In *E. coli* cells growing in poor S medium, *θ*_d_ exhibited a 1.5-fold decrease, while the fraction of constrained patches almost doubled relative to rapidly growing cells (Fig. 3a,b, Supplementary Fig. 10b,c and Supplementary Table 3). The fraction of MreB patches exhibiting random diffusion was remarkably constant in all strains and conditions, for both *B. subtilis* and *E. coli* (Fig. 3a, Supplementary Fig. 10a and Supplementary Table 3).

### MreB patch speed is proportional to growth rate in *B. subtilis*

We next extracted the kinetic parameters of MreB movements by fitting the MSD curves to different physical models^48^ (see Supplementary Methods and Supplementary Fig. 8). Second-order polynomial fit of MreB and Mbl-directed motions in single *B. subtilis* cells growing in LB (Supplementary Fig. 9a) revealed that patches exhibiting directed motion moved at an average speed (*ν*) of ∼55 nm s^−1^, consistent with the constant velocity obtained from the slopes of kymograph traces of circumferentially moving patches^31^ (Fig. 3c, Supplementary Fig. 7a and Supplementary Table 3). Like for patch density, patch speed was not noticeably affected by protein levels^31^,^32^ (Supplementary Fig. 6b and Supplementary Table 4). Linear fitting for MreB patches freely diffusing in the membrane (Supplementary Fig. 9b) revealed an apparent diffusion coefficient (*D*) of ∼0.001–0.003 μm^2^ s^−1^ (Supplementary Table 3). Complementary analysis of trajectories using cumulative distribution functions^49^,^50^ gave a similar value (*D* ∼0.001–0.005 μm^2^ s^−1^), which is comparable to the value obtained for the flagellar motor in the cytoplasmic membrane of *E. coli*^51^.

Do directed MreB patches change their speed in response to different growth media? Our ensemble analysis confirmed our previous observation^31^ that circumferentially motile MreB and Mbl patches displayed a broad distribution of speed across different cells (Fig. 3c) and even within the same *B. subtilis* cell (Supplementary Fig. 5d). Even so, the average speed of MreB and Mbl patches almost doubled in rich (LB) medium compared to poor (S) medium (Fig. 3c, Supplementary Fig. 5d, Supplementary Movies 1 and 2 and Supplementary Tables 3 and 4), suggesting that speed was dependent on growth rate. To confirm this, we imaged MreB and Mbl patches in two additional media, CH-rich medium and M9SE poor medium, in which *B. subtilis* displays different growth rates (Supplementary Movies 3 and 4 and Supplementary Tables 1 and 2). These results indicated that the speed of MreB/Mbl rotation is linearly proportional to nutrient-imposed growth rate in *B. subtilis* (Supplementary Fig. 11 and Supplementary Table 4).

In contrast, in *E. coli* the average speed of directed patches almost did not vary between rich and poor medium (Fig. 3c, Supplementary Movie 5, Supplementary Fig. 5d and Supplementary Table 3). This is consistent with the previous report of van Teeffelen *et al*.^33^, where velocity of mobile MreB patches in *E. coli* was found to be similar in LB and two M63-based minimal media. We nevertheless found a very slight but statistically significant decrease of patch speed (∼1.2-fold) in slowly growing cells relative to fast-growing cells (Fig. 3c), which suggests a mild adjustment of this parameter in different growth conditions.

### *B. subtilis* and *E. coli* adapt differently during nutrient upshift

**Figure 4:**
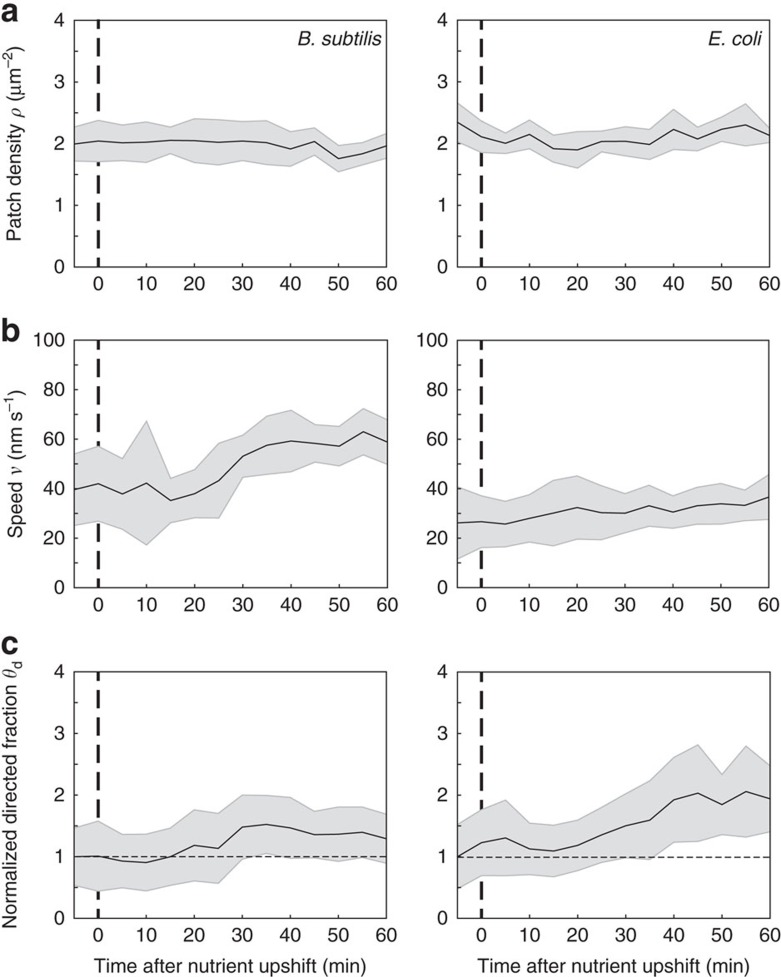
MreB patch regulation profile during nutrient upshift. Changes in MreB patch dynamics during nutrient upshift from S to LB medium in perfusion-based microfluidic channels at 37 °C. (**a**) Total patch density, *ρ*. (**b**) Directed patch speed, *ν*. (**c**) Normalized fraction of directed patches, *θ*_d_. Time is in minutes. Solid lines indicate average values from a minimum of 20 cells for *E. coli* and 50 cells for *B. subtilis*. Grey shaded areas indicate s.d. *n*=29–60 for *B. subtilis*, *n*=16–30 for *E. coli.* See Supplementary Fig. 13 for the concomitant adaptation profiles of cell dimensions, cell area and growth rate. Data are a compilation of at least two independent experiments.

Our results show that at steady-state *B. subtilis* adapts to different growth rates by modulating the speed of MreB patches while *E. coli* modulates almost imperceptibly both the speed and the fraction of directed MreB patches. However, it remained unknown how cells reach new steady states during growth rate shifts. Cells could adopt the same adaptation strategy or exhibit alternative or mixed regulation patterns during the transition. We used phase contrast and TIRFM to monitor growth and MreB patch properties (*ρ*, *ν* and *θ*_d_) in perfusion-based microfluidics channels during nutrient upshift, from S to LB (Supplementary Movies 6 and 7). Cell segmentation based on phase-contrast images was used to measure the surface area and the dimensions (length and width) of individual cells (Supplementary Fig. 13), as well as the growth rate at the single micro-colony level (Supplementary Fig. 13c) (see details in Supplementary Methods). In both organisms, cells kept growing as judged by the progressive increase of cell area right after media shift, without any lag (Supplementary Fig. 13c). *B. subtilis* cells increased their length and maintained a constant width, whereas *E. coli* cells started increasing their width immediately after nutrient shift (Supplementary Fig. 13b and Supplementary Movies 6 and 7). Importantly, MreB patches exhibited dynamic changes that were consistent with the adaptation profiles observed in steady-state growing cells. In both *B. subtilis* and *E. coli*, total patch density (*ρ*) remained constant throughout the nutrient shift (Fig. 4a). However, in *B. subtilis* the speed of mobile MreB patches (*ν*) rapidly increased ∼15 min after the shift (Fig. 4b). In contrast, *E. coli* gradually increased (up to ∼twofold) the fraction of directed patches (*θ*_d_) (Fig. 4c) and slightly but significant increased patch speed (Fig. 4b) right after the change of medium. This contrasting regulation of MreB dynamics mirrored significant differences in growth rate adaptation between the two organisms: growth rate immediately and rapidly increased in *E. coli*, while it displayed a ∼15 min lag before increasing in *B. subtilis* (Supplementary Fig. 13c). We argue that these different adaptations reflect different mechanisms of cell wall expansion in the two organisms (see Discussion section).

### Cell expansion per patch is constant in *B. subtilis*

**Figure 5:**
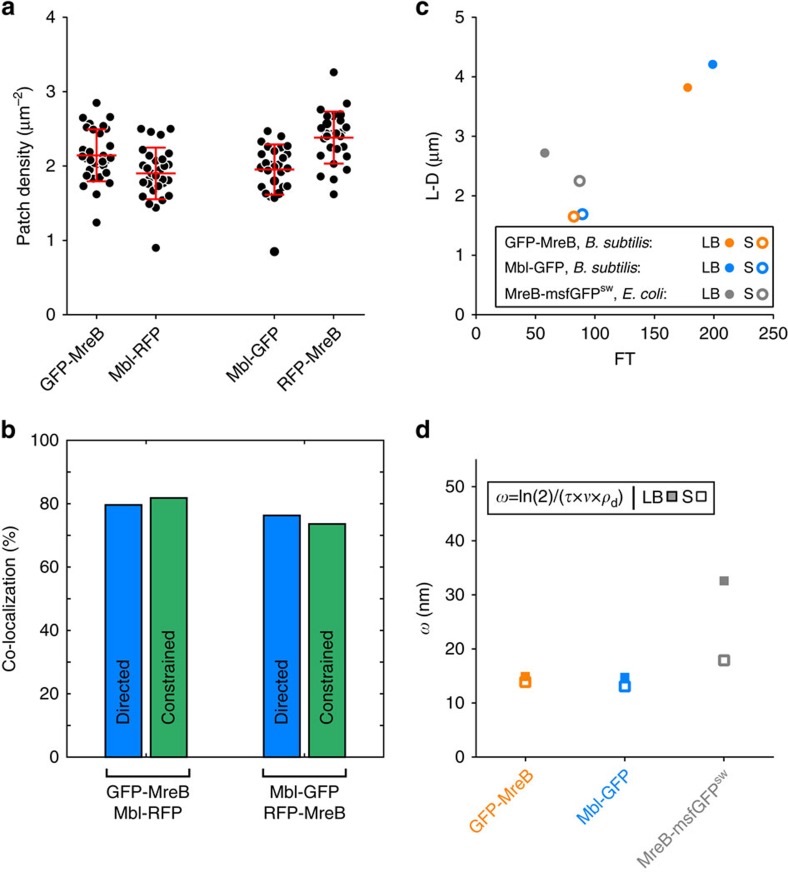
Numerical models link MreB-mediated PG insertion and extension of the cylindrical sidewall. (**a**) Patch density in *B. subtilis* co-localization strains expressing GFP-MreB and Mbl-RFP (strain RWSB18, *n*=30) or Mbl-GFP and RFP-GFP (strain ABS2408, *n*=27). Plotted averages and s.d.'s are shown in red. (**b**) Co-localization of GFP-MreB/Mbl-RFP and of Mbl-GFP/RFP-MreB in directed and constrained patches. Data are a compilation of at least two independent experiments. (**c**) Plot of average length of the cylindrical sidewall (cell length (*L*)—cell diameter (*D*), *L*−*D*) against putative full turns covered by directed MreB patches (FT) in single *B. subtilis* and *E. coli* cells grown in LB and S media. (**d**) Estimated average width of the PG bands inserted per MreB patch (*ω*) for *B. subtilis* and *E. coli* in LB and S media (see Supplementary Note 1 for details).

To verify our assumption that MreB patches are a proxy for PGEM synthetic activity, we wondered if we could correlate patch dynamics with overall sidewall extension during growth. The prevailing model assumes that only circumferentially mobile MreB patches are associated to active PGEMs and thus responsible for the insertion of new glycan strands along the cell cylinder in radial bands^25^,^52^,^53^, which is consistent with the current model of PG arrangement in rod-shaped bacteria^54^. We previously showed that in *B. subtilis* most of MreB and Mbl co-exist in motile patches using kymographs analysis^31^. Two-colour TIRFM on pairs of native and inducible fluorescent protein fusions showed that both MreB and Mbl display a patch density of ∼2 patches μm^−2^ in the co-localization strains too. We used symmetric permutations of GFP and mRFPruby (RFP) fusions to overcome the bias introduced by the weaker fluorescence emission emanating from the RFP fusions (Fig. 5a). SPT computational analysis confirmed that MreB and Mbl co-localize extensively in rotating patches, as well as in static patches (Fig. 5b). We can therefore assume in our model that MreB and Mbl are associated to the same PGEMs. We first calculated the theoretical PG elongation required for each generation, using cell length (*L*) and diameter (*D*) to obtain the average length of the cylindrical sidewall (*L*−*D*). Then, we used the measurable parameters of number of directed patches per cell (*N*_d_), patch speed (*ν*) and generation time (*τ*) to calculate the number of putative full turns that MreB patches do over one cell cycle (see Supplementary Note 1). Over one doubling time, *N*_d_ moving at a linear speed *ν* would cover a distance *N*_d_ × *ν* × *τ*. The number of full turns (FT) around the cell perimeter would then be FT=(*N*_d_ × *ν* × *τ*)/(*πD*). Our calculation gives between 60 and 200 full turns per generation time depending on the condition tested (Supplementary Note 1 and Supplementary Table 8). Next, we wondered whether larger cell length extension correlates with larger number of putative MreB full turns. We plotted (*L*−*D*) against FT, and observed a linear relationship between the two parameters for *B. subtilis*, but not for *E. coli* (Fig. 5c), indicating that only in *B. subtilis* there is a direct relationship between cell elongation and MreB dynamics.

Based on the idealized model where glycan strands run perpendicular to the long axis of the cell, and on the assumption that MreB and Mbl always co-localize in *B. subtilis*, we extracted the average width *ω* of the new PG band inserted (that is, increment on axial length) per full turn of MreB patches (*ω*=(*L*−*D*)/FT). Interestingly, a constant *ω* was found in *B. subtilis* irrespective of the fusion and growth medium, while *ω* almost doubled in *E. coli* between poor and rich medium (Supplementary Note 1 and Supplementary Table 9). A more refined calculation of *ω* based on the integration of PG synthesis over the cell cycle (*ω*=ln(2)/(*τ* × *ν* × *ρ*_d_), see Supplementary Note 1 and Supplementary Table 9) showed a constant *ω* of ∼14 nm in *B. subtilis* in all conditions versus ∼18 nm and ∼31 nm for slowly and rapidly growing cells, respectively, in *E. coli* (Fig. 5d and Supplementary Table 9). Thus, our numerical models linked MreB-mediated PG synthesis and cell elongation, providing mechanistic insight into nutrient-dependent control of cell size in rod-shaped bacteria (see Discussion section).

## Discussion

It has been appreciated for more than half a century that cell size of bacteria varies substantially under different nutrient conditions. However, it is only after the discovery of bacterial tubulin and actin homologues, in combination with advanced imaging platforms, that investigation of the molecular mechanisms underlying cell size regulation was made possible^47^. In this study, we monitored MreB patches as a proxy for PGEM-mediated sidewall elongation and found shared as well as distinct traits in *B. subtilis* and *E. coli*.

In steady state, the number of MreB patches linearly increased throughout the cell cycle, consistent with our previous report that there is a correlation between the number of MreB tracks and cell length^31^. This indicates that in a given growth medium MreB-associated PG-synthesizing activity per unit of surface area remains constant during the cell cycle, suggesting that both *B. subtilis* and *E. coli* cells follow exponential growth at the single-cell level. A recent report reaches a similar conclusion by measuring cell mass at exceptional precision^55^.

**Figure 6:**
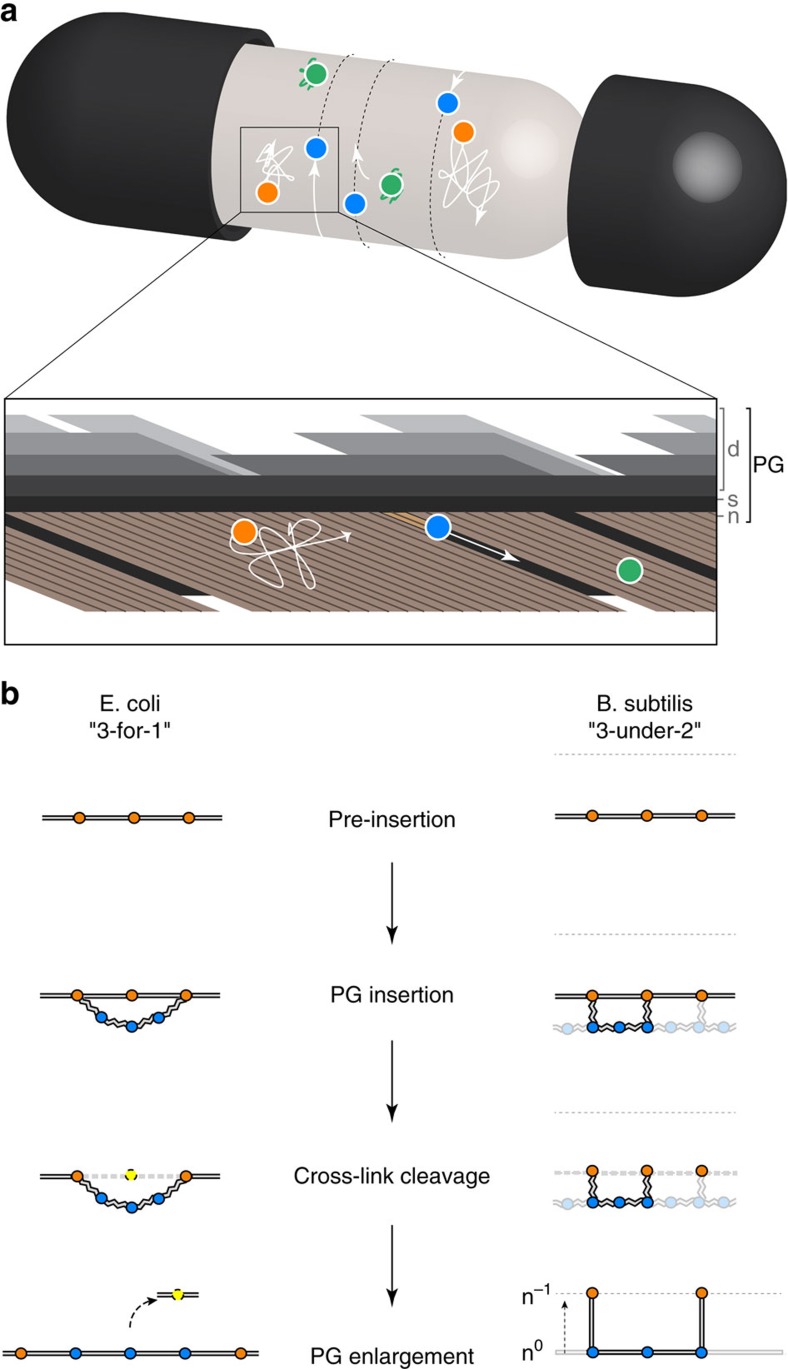
Models of PGEM-mediated insertion of PG in rod-shaped bacteria. (**a**) Schematic illustration of MreB patches exhibiting different dynamic behaviours in the cytoplasmic membrane. Orange dots, randomly diffusing complexes; blue dots, directionally moving complexes; green dots, complexes in stand-by. A Gram-positive bacterium is represented (see Supplementary Fig. 13 for a comparative illustration of Gram-positive and Gram-negative bacteria). Zoom-in, view from inside the cell. PG, peptidoglycan; d, discontinuous outermost PG layers; s, stress-bearing PG layer; n, newer, uncompleted innermost PG layer. (**b**) Models for PG insertion at the molecular level. In *E. coli*, according to the prevailing model proposed by Höltje *et al*.^8^, one existing glycan strand is replaced by three new glycan strands (‘3-for-1' model) using a ‘make-before-break' strategy: (i) a triplet of newly synthesized glycan strands in a relaxed state is hooked underneath a single strand, (ii) the triplet is covalently linked to the sacculus by transpeptidation of the two outer strands, (iii) on release of the old strand, the newly added triplet is automatically pulled into the existing layer due to surface tension. As a result, the sacculus expands by two peptide bridges. In *B. subtilis*, we propose that three new glycan strands are inserted underneath two existing strands of the innermost PG layer (n^0^) used as template (‘3-under-2' model): (i) a triplet of newly synthesized glycan strands is hooked in a relaxed state underneath two preexisting strands of the stress-bearing innermost layer of the sacculus and (ii) linked by transpeptidation of the two outer strands; (iii) following an ‘inside-to-outside' growth strategy, the newly added triplet becomes stress bearing as the older layer (n^−1^) is stretched following its peptide brides degradation. As a result, the length of the sacculus expands by one peptide bridge distance. Circles represent glycan strands. Blue, orange and yellow indicate newly synthesized, preexisting and degraded glycan strands, respectively. Straight, winding and dashed grey lines indicate stretched, relaxed and degraded, respectively, peptide bridges.

Computational analysis revealed that in exponentially growing *B. subtilis* and *E. coli* cells only a fraction of all MreB patches exhibit canonical circumferential motion. This fraction is constant in all conditions (growth rates) tested for *B. subtilis*, and appears slightly dependent on growth rate in *E. coli*. Previous studies focused on these motile patches; only in a few instances non-mobile patches were reported without further analysis^33^,^42^ or were attributed to some loss of normal MreB function^56^. Similar to a recent report that used SPT analysis to distinguish different ribosome states in *E. coli* cells^57^, we propose that MreB patches exhibiting diverse MSD curves might be in distinct physiological states (Fig. 6a and Supplementary Fig. 14). Consistent with this idea, we observed patch trajectories exhibiting rapid transition between modes of movement (Supplementary Fig. 9d). It is conceivable that randomly diffusive MreB patches scan the cell inner membrane to identify cues related to areas requiring new PG insertion. Accordingly, local bending of the cell surface has been proposed to drive MreB localization in *E. coli*^42^. Patches exhibiting constrained diffusion could reflect complexes recruiting missing components of the PGEM to initiate local PG insertion and/or stalled complexes. Finally, the processive activity of recruited PG glycosyltransferases^58^,^59^ would drive the circumferential motion of MreB patches. Nonetheless, we cannot exclude the possibility that diffusive MreB patches reflect new PG insertion too. In this case, the fact that the density of MreB patches exhibiting random diffusion was remarkably constant in all strains and conditions for both *B. subtilis* and *E. coli* (Supplementary Fig. 10a) suggests however that such MreB-associated diffusive PG synthesis would not be regulated in response to nutrient availability. It was recently reported that PG polymerization also occurs in a diffusive manner, outside MreB-associated PGEMs, by the action of class A penicillin-binding proteins (aPBPs)^34^, ^41^. aPBPs and PGEM systems were suggested to be partially interdependent, collaborating with each other at some level to promote PG biogenesis^34^. Since MreB has been reported to be associated and to interact with aPBPs^45^,^60^,^61^, it remains plausible that the fraction of MreB exhibiting random diffusion is associated to aPBPs-dependent diffusive PG synthesis. In this scenario, our results would be consistent with the hypothesis put forward by Cho *et al*.^34^ that the more broadly conserved PGEM system might build the primary structure of the PG scaffold (and thus be regulated to enable varied growth rates and sizes) while the aPBPs system may fill in gaps that arise during PG expansion and/or damage.

How might the properties and the localization of MreB assemblies be regulated? Drawing parallel to eukaryotic actin, it is tempting to speculate that differences in polymerization kinetics might modulate MreB behaviour. It is also possible that proteins or metabolites interacting with MreB directly influence MreB localization and/or regulate the size and geometric structure of MreB oligomers, like actin-binding proteins in eukaryotic cells^62^,^63^. Consistently, the transmembrane protein RodZ was recently shown to couple MreB to the rotating PGEMs in *E. coli*, and it was suggested that MreB maintains its ability to initiate the sites of new cell wall synthesis, in a rotation-independent manner, either directly or through a second-linker protein^43^.

Our work provides several lines of evidence for regulation of MreB patches in the cell: (i) patch number linearly increases throughout the cell cycle (that is, total patch density remains constant); (ii) there are subpopulations of patches with distinct dynamics; (iii) patch speed is dependent on growth rate in *B. subtilis* and (iv) both patch speed and the fraction of directed MreB patches vary slightly at different growth rates in *E. coli*, but these changes alone cannot account for the corresponding differences in cell size. What could we infer from these results regarding the mechanism of MreB-mediated PG insertion at the molecular level? The dynamics and the 3D structure of Gram-negative and Gram-positive cell walls are poorly understood but their monolayered and multilayered nature, respectively, necessarily involves different modes of growth. In rod-shaped bacteria, the prevailing ‘3-for-1' model, which hypothesizes that three new glycan strands replace one preexisting strand per insertion event (Fig. 6b and Supplementary Fig. 15b), satisfactorily explains Gram-negative sacculi elongation^8^. This model would account for the observed high rate of PG turnover in *E. coli*^8^, and supports a perfectly sustainable and safe sidewall enlargement. However, this mechanism cannot explain the maintenance of a multilayered rod-shaped sacculus through generations and can therefore not be applied to Gram-positive bacteria. Several assumptions need to be made to account for the generation and maintenance of a thick, multilayered cell wall, and to our knowledge no model has been proposed so far. Here we propose a simple mechanism of Gram-positive sidewall enlargement, namely the ‘3-under-2' model (see Supplementary Note 2 for a detailed description). In brief, our model relies on the two following assumptions: (1) as proposed before^8^,^64^,^65^,^66^, concentric multilayered Gram-positive cell walls follow an ‘inside-to-outside' mode of growth; and (2) the newly generated -hence innermost- layer binds the previous layer, which is used as scaffold, with a 3:2 geometry (Fig. 6b, Supplementary Fig. 15c and Supplementary Note 2).

Strikingly, the width *ω* of the new PG band inserted per MreB patch in both *B. subtilis* and *E. coli* only differs by less than an order of magnitude from the estimated PG unit length (estimated width of a stretched sugar strand plus a peptide crosslink, ∼4–5 nm (ref. 67)). This indicates that we are not significantly undercounting the number of directed patches even though our ‘well fit' directed motions are likely to be low estimates due to natural noise within tracking data and to mixed patterns of motion. Conciliation of our numerical models and the theoretical ‘3-for-1' and ‘3-under-2' models of PG insertion in *E. coli* and *B. subtilis*, respectively, suggests that in *B. subtilis* one MreB patch might coordinate, regardless of the growth rate, the insertion of two triplets of glycan strands, either by the action of one PGEM, or of several PGEM complexes associated to the same patch. Notably, our ‘3-under-2' model of growth is compatible with tandem insertion of more than three new glycan strands (Fig. 6b, Supplementary Fig. 15c, Supplementary Note 2 and Supplementary Table 10). In contrast, in *E. coli*, one MreB patch might coordinate the insertion of a variable number of triplets of glycan strands depending on the growth rate (that is, two in S medium and three–four in LB medium).

The orthogonal regulatory mechanisms reported here may reflect the different ultrastructure and integrity constraints of Gram-positive and Gram-negative cell walls. The multilayered sacculus of *B. subtilis* imposes that an entire new layer of PG is produced per generation. In contrast, insertion of new PG units could happen anywhere along the monolayered sidewall of *E. coli*. A direct consequence of this is that spatial control of PG insertion sites must be tighter in *B. subtilis* than in *E. coli*. This may be a cause for the constant amount of PG inserted per patch, regardless of the growth conditions, while this parameter appears less constrained in *E. coli*. It is also tempting to speculate how different mechanisms of new PG insertion might influence regulation of MreB-mediated PGEM activity. Our finding of contrasting characteristics of MreB properties in *B. subtilis* and *E. coli* provides key clues to understand the basic organizational principles of Gram-positive and Gram-negative cell envelope. Future work will provide further insights into the structure–function relationship of MreB assemblies, and investigate the ‘3-under-2' model for the growth of Gram-positive sacculi proposed here. Super-resolution imaging techniques will also be needed to elucidate the ultrastructure of MreB patches below the diffraction limit. We envision that these studies will reveal a yet more complex picture of the spatiotemporal organization of individual MreB assemblies.

## Methods

### General methods and bacterial growth conditions

Methods for growth of *B. subtilis*, transformation, selection of transformants and so on have been described extensively elsewhere^68^. DNA manipulations were carried out by standard methods. *B. subtilis* and *E. coli* strains were grown at 30 or 37 °C in rich lysogeny broth medium (LB), casein hydrolysate medium (CH)^68^, S (ref. 3) or M9SE (ref. 69) medium supplemented with 25 mM MgSO_4_, 1 mM isopropyl-β-D-thio-galactopyranoside, 0.5% xylose and/or antibiotics when indicated. Antibiotics were used at the following concentrations: chloramphenicol, 5 μg ml^−1^; kanamycin, 10 μg ml^−1^; spectinomycin, 100 μg ml^−1^; erythromycin, 1 μg ml^−1^. *B. subtilis* and *E. coli* strains used in this study are listed in Supplementary Table 5. Plasmids are listed in Supplementary Table 6. The sequences of oligonucleotides used are listed in Supplementary Table 7. The constructions of a strain inactivated for *mbl*, and a strain expressing a fusion of *mreBH* to a SPA-tag are described in the corresponding sections of the Supplementary Methods.

### TIRFM

Time-lapse TIRFM movies were taken on at least two different days for each strain and condition. Cells were first grown in shaking flasks or U-bottom 96-well cell culture plates (CellStar) at 37 °C to reach early exponential phase (OD_600_∼0.1). One microlitre of the liquid culture was spotted onto a thin agarose pad (1%), topped by a coverslip and immersion oil, and mounted immediately in the temperature-controlled microscope stage. All experiments were done inside the incubation chamber at 37 °C, within 10 min after taking the sample. For all GFP fusions exposure time was 100 ms. Inter-frame intervals were 1 s over 2-min movies. Imaging was performed on an inverted microscope (Nikon Ti-E) with an Apo TIRF × 100 oil objective (Nikon, NA 1.49), with either diode-pumped solid-state lasers (Cobolt Calypso, 50 mW, 491 nm and Cobolt Jive, 50 mW, 561 nm) or an iLas2 laser coupling system from Roper Scientific (150 mW, 488 nm and 50 mW, 561 nm). Images were collected with an electron-multiplying charge-coupled device camera (iXON3 DU-897, Andor) at maximum gain setting (300) attached to a × 2.5 magnification lens. Final pixel size was 64 nm. Image acquisition was controlled by the NIS-Elements (Nikon) or Metamorph v.7 software packages.

### Detection of MreB patches

Movies containing single cells were cropped individually out of raw images of full fields of view (Supplementary Fig. 4a). On single-cell movies, image analysis was performed to detect patches on each frame of the time series and to link these localizations on consecutive frames. All computations were implemented in MATLAB (Mathworks, R2014b). MreB patch identification was then performed in several steps (Supplementary Fig. 4b–e). First, a MIP image was created. Second, the MIP image was converted into a binary image based on threshold determined using the Otsu's method^70^, allowing to identify cell contour and to measure cell area (number of pixels within the cell contour multiplied with pixel area). Third, MreB patches were identified in each frame of the time series using the comet detection approach of the u-track 2.1.3 software (MATLAB-based package developed in the Danuser lab^71^,^72^). Object detection relied on enhancement of raw images (difference of Gaussian, *σ*_1_=1 pixels and *σ*_2_=4 pixels) to remove high-frequency intensity fluctuations and reduce cell background, followed by watershed-based segmentation (minimum threshold=4 s.d. of image intensity, with a step size=1 s.d.) to extract the coordinates of each object. Patch detection was confirmed by visual examination. Last, MreB patch number was calculated as the average number of patches detected over all frames in each single-cell movie (Supplementary Fig. 5a–c), and divided by cell area to obtain patch density. The minimum width of patches was estimated to be ∼300 nm (see Supplementary Methods), and the average distance between closest neighbouring patches was >600 nm, indicating that patch density is limited to 4–8 μm^−2^ (theoretical density, assuming the perfect localization of patches onto a flat squared grid). Alternative algorithms based on local maxima in a small window size or on Gaussian fitting provided comparable results for patch density, ensuring the robustness of our detection.

### SPT

SPT was performed using u-track by linking localizations close to each other on consecutive frames, assuming that particles exhibit random motion (minimum Brownian search radius of 0 pixels and maximum Brownian search radius of 3 pixels) or directed movement. The motion model for each particle was determined from its track using at least five frames. No missing link was allowed in the tracking (Maximal Gap to Close=0), and splitting or merging of tracks were also not allowed, meaning that linking cost during reconnection were mainly dependent on patch-to-patch distance. Supplementary Figure S7b shows an example of two close MreB patches that were well separated into two different trajectories (red and yellow). To ensure accuracy of MSD analysis and subsequent derivation of patch speed, only trajectories consisting of no less than four steps were selected for speed calculation. Equivalent analysis using traces with minimal lifetimes of eight frames yielded similar values (Supplementary Table 3).

### Classification of MreB patch dynamics

MSD of all trajectories were obtained by calculating 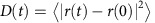, where *r* is the position of the tracked particles and *t* the time interval (*t* being no longer than the 7/8 of the full length of each track). Next, MSD curves were subjected to fittings of both directed motion (MSD(*t*)=(*νt*)^2^) and random diffusion (MSD(*t*)=4*Dt*). Determination of the mode of movement was based on the coefficient of determination (*R*^2^) obtained with the fittings: directed (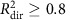 and 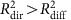), random (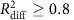 and 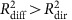) (Supplementary Fig. 8i). If *R*^2^ of both fittings was below 0.8 and the maximum MSD value <0.05 μm^2^, the trajectory was categorized as constrained diffusion. Patches that could not be well fitted (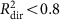 and 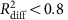 and MSD >0.05 μm^2^) were too ambiguous to be accurately classified according to the limits set up by the temporal and spatial resolution of our measurements and were therefore assigned to the unclassified category. Values used to classify the mode of movement were based on simulations of random walks and directed movements (in 2D) and on control experiments with fixed *B. subtilis* cells expressing GFP-MreB (see details in Supplementary Methods). Finally, each patch detected was assigned to a class (directed, random, constrained or unclassified) for all frames of its corresponding movie, allowing to calculate instantaneous patch density and mobile fractions per frame, and thus to estimate averages at the single-cell level. Only trajectories exhibiting directed motion were used to obtain average speed (*ν*). Errors in the distribution (%) of MreB patches among the four classes and on the density and speed of directed patches were estimated through bootstrap samples of single tracks and showed low s.e. (∼5% in relative) (see Supplementary Table 4).

### MreB and Mbl co-localization using SPT and kymograph analysis

For two-colour TIRFM experiments, a dual band filter set was used. Exposure time was 200 ms and frame rate 1 s. We verified the absence of bleed-through and cross-talk over the two channels and the absence of chromatic aberration using 0.1 μm diameter beads stained with several fluorescent dyes (TetraSpeck microspheres, Invitrogen). SPT of patches was performed in the green channel, because signal-to-noise ratio in the red channel was too weak for this purpose, and the resulting trajectories were subjected to MSD analysis to identify directed and static patches. Cells were also segmented on the bright-field image to obtain their longitudinal axis. To generate kymographs on the green and red channels, lines were drawn either perpendicular to the longitudinal axis and intercepting the average positions of static patches or based on linear regression of directed patches trajectories. Finally, all kymographs were inspected to quantify MreB and Mbl co-localization.

### Cell growth and patch dynamics during media upshift

*B. subtilis* and *E. coli* growth during media shift were monitored under a Nikon TI microscope using a microfluidics flow chamber (CellASIC, EMD Millipore), allowing to follow growth and division at the single-cell level for several generations. *B. subtilis* cells carrying GFP-Mbl and *E. coli* cells carrying MreB-msfGFP^SW^ were grown in S medium at 37 °C into CellASIC plates for 60 min. S medium was then replaced by LB, and cells were allowed to grow for at least an additional hour. Bright-field and TIRFM images were acquired 5 min before, right after (*t*=0) and every 5 min after the nutrient upshift. TIRFM acquisition parameters and SPT analysis were as described for the measurements under steady-state growth. See Supplementary Methods for more details.

### Data availability

The authors declare that the relevant data supporting the findings of this study are available within this paper and its Supplementary Information Files, or from the corresponding author on request.

## Additional information

**How to cite this article:** Billaudeau, C. *et al*. Contrasting mechanisms of growth in two model rod-shaped bacteria. *Nat. Commun.* **8,** 15370 doi: 10.1038/ncomms15370 (2017).

**Publisher's note:** Springer Nature remains neutral with regard to jurisdictional claims in published maps and institutional affiliations.

## Supplementary Material

Supplementary Information Supplementary Notes, Supplementary Methods, Supplementary Figures, Supplementary Tables and Supplementary References

Supplementary Movie 1 Movies of representative exponentially growing B. subtilis cells expressing GFP-MreB in LB and S media at 37 °C. Maximum intensity projection is included as the last frame. Exposure time: 100 ms.

Supplementary Movie 2 Movies of representative exponentially growing B. subtilis cells expressing Mbl-GFP in LB and S media at 37 °C. Maximum intensity projection is included as the last frame. Exposure time: 100 ms.

Supplementary Movie 3 Movies of representative exponentially growing B. subtilis cells expressing GFP-MreB in CH and M9SE media at 37 °C. Maximum intensity projection is included as the last frame. Exposure time: 100 ms.

Supplementary Movie 4 Movies of representative exponentially growing B. subtilis cells expressing Mbl-GFP in CH and M9SE at 37 °C. Maximum intensity projection is included as the last frame. Exposure time: 100 ms.

Supplementary Movie 5 Movies of representative exponentially growing E. coli cells expressing MreB-msfGFPSW in LB and S media at 37 °C. Maximum intensity projection is included as the last frame. Exposure time: 100 ms.

Supplementary Movie 6 Phase-contrast time-lapse imaging of representative exponentially growing B. subtilis cells in response to nutrient upshift from S to LB media at 37 °C. t=0 indicates medium switch.

Supplementary Movie 7 Phase-contrast time-lapse imaging of representative exponentially growing E. coli cells in response to nutrient upshift from S to LB media at 37 °C. t=0 indicates medium switch.
