# Supplementary material for: Contrasting mechanisms of growth in two model rod-shaped bacteria
Source: Nat Commun. 2017 Jun 7;8:15370. doi: 10.1038/ncomms15370 (PMC5467245; doi:10.1038/ncomms15370)
Supplement: Supplementary Information — Supplementary Notes, Supplementary Methods, Supplementary Figures, Supplementary Tables and Supplementary References [file ncomms15370-s1.pdf]

Supplementary Note 1. Integration of cell wall synthesis along the bacterial cell cycle.

The amount of PG produced by the PG elongation machineries (PGEMs) associated to MreB patches cannot be directly calculated from our data because of the uncertainty concerning the number of glycan strands inserted per patch (estimated width of a cross-linked glycan strand, ~ 4.5 nm; to be compared to patches close to the diffraction limit, < 500 nm)[1]. However, it is possible to calculate the theoretical surface of PG required to double the length of the cell over one generation, which divided by the distance travelled by all MreB patches in the cell would allow to deduce the average width of the PG band inserted by each individual patch.

Estimation of the theoretical PG elongation required per generation. The theoretical elongation of the sacculus required to double the length of the cell over one generation should equal the length of the cylindrical sidewall. Using the average length (L) and diameter (D) of the cell, then the average length of the sidewall is $(L - D)$. This corresponds to the apparent elongation, without making any assumption on the mechanism driving this elongation.

Calculation of the number of full turns around the cell circumference that mobile MreB patches do over one cell cycle. Using circumferentially moving MreB patches as a proxy for PG synthesis, the number of putative full turns (FT) travelled by PGEMs during one cell cycle can be calculated from the generation time (τ) and the measurable number (N_d) and speed (ν) of directed patches per cell. N_d is the product of directed patch density (ρ_d) and cell surface ($S = D(L - D)$). ρ_d was itself determined as the ratio of the number of directed patches in the TIRF field of view by the area of this field. Over one doubling time, mobile patches moving at a linear speed ν would cover a distance:

$$N_d \times \nu \times \tau.$$

FT around the cell perimeter (πD) would then be:

$$FT = (N_d \times \nu \times \tau)/(\pi D).$$

Thus, FT around the cell perimeter would vary from be 82-90 (for MreB and Mbl respectively) in poor S medium to 178-199 in rich LB medium in *B. subtilis*. Conversely, MreB in *E. coli* would decrease from 87 FT in S to 58 in LB medium (See Supplementary Table 8).

<u>Calculation of the average width of the PG band inserted per patch per cell cycle</u>. As a first approximation, the width ω of the PG band inserted per MreB patch can be expressed as the ratio of the theoretical PG extension required per cell cycle and the number of full turns:

$$\omega \ = \ (L - D) \ / \ FT.$$

<u>Refined calculation of the average width of the PG band inserted per patch per cycle</u>. A more refined calculation of ω can be implemented based on the integration of PG synthesis over the cell cycle. On a small interval of time, each PGEM synthesizes a given length of glycan strand, which leads to a local increase of cell length. For an interval of time δt, the sum of the contributions of all active PGEMs leads to a total increase of cell length $\delta l(t)$ given by:

$$\delta l(t) \ = \ (\omega \times N_d(t) \times \nu(t) \times \delta t)/(\pi D), \quad (*)$$

We now introduce $l(t)$ as the length of the cell cylinder where PG synthesis is active. Since the poles have a hemispherical shape then $l(t)$ and $L(t)$ are related by this simple relation:

$$l(t) \ = \ L(t) - D.$$

Assuming that δt is small enough, we can then deduce from expression (*) the following differential equation:

$$dl(t)/dt \ \approx \big(l(t + \delta t) - l(t)\big)/\delta t = \delta l(t)/\delta t = \big(\omega \times N_d(t) \times \nu(t)\big)/(\pi D) \qquad (**)$$

where $dl(t)/dt$ denotes the time derivative of the length of the cell cylinder at the time t. We found that for both *B. subtilis* and *E. coli*, ρ_d remains constant for a given growth rate, therefore:

$$N_d(t) = l(t) \times \pi D \times \rho_d$$

Expression (**) can then be rewritten as $dl(t)/dt = \omega \times \nu \times \rho_d \times l(t)$. We then deduce that for every t in $[0, \tau]$ we have $l(t) = l_0 \exp(\omega \times \nu \times \rho_d \times t)$ where l_0 is the length of the cell cylinder at birth. By definition, for $t = \tau$, we have $l(\tau) = 2l_0$ and then $\omega \times \nu \times \rho_d \times \tau = \ln(2)$ which can be rewritten as $\omega \times \nu \times \rho_d = \mu$, where μ is the growth rate. Consequently, for a given growth rate μ, we have:

$$\omega[\mu] = \ln(2)/(\tau[\mu] \times \nu[\mu] \times \rho_d[\mu])$$

where all the constants depend possibly on the growth rate μ.

Using the two methods described above, we can estimate the width of the PG band inserted per patch, ω (in nm), for each MreB fusion and growth medium (See Supplementary Table S9).

Supplementary Note 2. Mechanistic models of cell wall synthesis in bacteria

Here, we propose a mechanistic model that describes PG synthesis in Gram-positive bacteria. The complexity of the multilayered cell wall of Gram-positive bacteria has, to our knowledge, prevented attempts in building mathematical or even mechanistic models so far. The mechanistic model that we propose is consistent with published observations and would allow to maintain a sustainable, thick PG meshwork in Gram-positive bacteria. For Gram-negative bacteria, we have retained the prevailing model in the field (as justified below).

<u>Assumptions of PG structure and insertion shared by both *B. subtilis* and *E. coli*.</u>

The detailed ultrastructure of the sacculus and the precise biochemical reaction(s) coupled to MreB-associated PGEMs are not yet established [2,3]. Here, we will assume that parallel <u>circumferential</u> glycan strands cross-linked by peptide bridges run perpendicular to the long axis of the cell [4], which is the prevailing model of PG organization (Supplementary Fig. 15a). We will also assume that *E. coli* has a monolayered PG (even though its thickness is compatible with 1 or 2 layers) and that *B. subtilis* has several concentric layers of PG [2].

Finally, and based on the early work of Pooley, Mobley, Koch, Doyle and others, we will presuppose an “inside-to-outside” mode of growth for the PG of *B. subtilis*, where new layers of PG are added to the innermost face of the sacculus, pushing outwards the previous layers, which will be eventually degraded, accounting for the observed PG turnover [5-9].

<u>Potential mechanism for growth of the monolayered PG meshwork of *E. coli*.</u>

In *E. coli*, several possibilities have been envisioned along the years to explain how new glycan strands are inserted in the PG layer: 1- the "cut and insertion" strategy from Burman and Park [10,11], where cleavage of the peptide bonds between 2 glycan strands are coordinated with insertion of one or two new strands (Supplementary Fig. 15b, left panel); 2- the "3-for-1" mechanism [11], which hypothesizes that three new glycan strands are co-inserted and replace one pre-existing strand, which is degraded (Fig. 6b and Supplementary Fig. 15b, right panel). Insertion of a single strand is not favored because its stem peptides could not face properly the stem peptides of the two adjacent strands, introducing stress on the cross-links. This was recently confirmed in the comprehensive study by Nguyen and co-workers in which a mathematical model describing PG synthesis in *E. coli* was generated [12]. Simultaneous insertion of 2 glycan strands should theoretically suppress this issue, as it does the "3-for-1" model (where there is a <u>net</u> insertion of 2 strands). This prediction was confirmed, and both modes of growth were shown to be compatible with robust cell wall increase in their model [12]. However, all “cut and insertion” strategies failed to explain the high rate of PG recycling per generation in *E. coli,* while the "3-for-1" model imposes that 50 % of the glycan strands are released during one cell cycle. We will therefore consider the "3-for-1" model as the most compelling in *E. coli*.

<u>Potential mechanism for growth of the multilayered PG meshwork of *B. subtilis*.</u>

The "3-under-2" model. In *B. subtilis*, insertion strategies such as the "3-for-1" mechanism do not apply because of the assumptions that the PG meshwork is multilayered and that growth

occurs in a push-up, inside-to-outside way. Insertion of glycan chains in a pre-existing layer would expand it laterally and quickly “dilute” potential upper layers, irrevocably leading to a monolayered PG and excluding an inside-to-outside mode of growth. Our two original assumptions impose a mechanism where at least one complete new layer of PG is generated per cell cycle, using the innermost pre-existing PG layer as scaffold. If a single PG layer is added per cell cycle and the cell has to double its length, then the simplest model is that newly inserted PG layers will have twice as many glycan strands than their template layer. In this scenario, every other glycan strand of the new layer will be connected to the upper (older) layer, in a “3-under-2” geometry (Fig. 6b and Supplementary Fig. 15c).

Expansion through hydrolysis of lateral cross-links. Addition of new PG layers will not allow expansion without a somewhat synchronized cleavage of peptide cross-links on the upper layers. Such cleavage will transfer the tension created by the osmotic pressure onto the newer, innermost layer(s) (containing twice as much strands and cross-links), thereby allowing local lateral expansion (Supplementary Fig. 15c). In our "3-under-2” model, we make the hypothesis that the stress-bearing layer is the innermost PG layer (Supplementary Fig. 15c, left panel), but it is also conceivable that an upper layer (or several layers) bears the stress (Supplementary Fig. 15c, right panel). In this situation, maximum expansion of the cell would be constrained by the uppermost stress-bearing layer, and the lower stress-bearing layer(s) could act as a safety net in case of potential injuries in the meshwork. They would also constitute a significant amount of material: if for example the 3rd layer bears the stress by being at its maximal extension, the 2nd layer would represent twice the surface of the cell and the 1st layer would compact 4 times the material needed to entirely cover the cell. In addition to obvious crowding issues (can a cell compact cross-linked glycan strands at 1.25 nm or even 2.5 nm distance?), there would also be an energetic cost for producing so many

“unused” glycan strands. We will therefore consider the most simple and thrifty case where only the innermost layer is stress-bearing (Supplementary Fig. 15c, left panel).

Mechanistic consequences of the “3-under-2” model on PGEMs and PG assembly.

The "3-for-1" model of PG insertion in Gram-negative bacteria is mechanistically simple. Three new glycan strands replace an old one. Whether transglycosylation occurs before transpeptidation or simultaneously would not change the mechanism [12]. However the "3-under-2" model for Gram-positive bacteria raises mechanistic questions regarding how the innermost new layer is connected to the scaffold, and this has important implications for the structure of the cell wall. Because three strands constitute the minimal geometrical unit connected to the upper layer, it is tempting to imagine that, like in the "3-for-1" model, there is co-synthesis or at least attachment of three glycan strands together (i.e. as a triplet) to the meshwork. A direct consequence of the 3-under-2 geometry is that addition of one triplet would enlarge the cell by one crosslink length only (Supplementary Fig. 15c, see also Fig. 6b), and that a cell should replace every single crosslink in order to double its length. This differs from the 3-for-1 mechanism, where only half the crosslinks need to be replaced per generation. Another consequence of this mechanism is that bundles of glycan strands could be co-inserted. While it is hard to imagine insertion/replacement of more than one triplet of glycan strands in the "3-for-1" model due to the local crowding, attachment to the upper layer could allow larger bundles of glycan strands to be inserted simultaneously in the "3-under-2" model.

Consequences of the mechanistic and mathematic models on the PGEMs

Our numerical data allowed to calculate the net (apparent) width of PG inserted per turn of MreB patch, $\omega[\mu]$, which derives from the net elongation of the sidewall (without any assumption on the mechanism driving this elongation). Based on the mechanistic models of PG insertion described above, it is then possible to speculate on the theoretical number of

glycan strands inserted per MreB patch. In *E. coli*, if the “3-for-1” model applies, one old glycan strand is replaced by three new glycan strands. While the net increase per insertion event would be two peptide crosslinks, the actual synthesis would be 1.5 x larger (three crosslinks). In this scenario, the number of glycan strands inserted should be 1.5 x bigger than the number deduced from the apparent width of the PG band inserted. A similar reasoning applies for *B. subtilis*. If we make the hypothesis that synthesis follows a "3-under-2 model", cells are producing per cell cycle twice the surface present at the beginning of the cycle. In other words, locally the actual PG produced must be twice the quantity deduced from the apparent extension.

Based on these models, the amount of PG inserted per MreB patch falls into a narrow window of 6 to 10 glycan strands for both organisms (See Supplementary Table 10). If true, this would suggest that MreB-associated PGEMs couple 2-4 triplets in average in both *E. coli* and *B. subtilis*. Considering the current resolution of MreB patches, both tandem side-by-side or distant insertion of triplets -potentially coordinated by the MreB polymers- are possible.

Supplementary Methods

Construction of a mutant strain (RCL78) inactivated for *mbl*

We noticed that the published *B. subtilis* strain 4261, a widely used *mbl* mutant [13], contained several unreported SNPs in *mbl-s*urrounding genes (namely *rapD*, *flhP* and *ywoH*), plus a deletion in *flhO*. This prompted us to construct a new *mbl* deletion mutant devoid of additional intergenic mutations by transformation/recombination of a PCR-ligated long flanking homology cassette into wild-type 168 *B. subtilis*. To this end, upstream and downstream regions of *mbl* were PCR-amplified using oligonucleotides RCL12/23 and RCL25/27, respectively and PCR-ligated with a chloramphenicol-resistance cassette amplified from pSWEET using primers RCL004/RCL005 [14]. The resulting DNA fragment was transformed into *B. subtilis* 168 and *mbl* minus recombinant clones selected on appropriate medium, giving strain RCL78. The subsequent complete sequencing of this strain revealed no additional mutations.

Construction of strains with *mreBH* fused to a SPA tag

Since anti-MreBH antibodies are not available, we created an *in situ* translational fusion between the 3' end of *mreBH* and a SPA sequence (*sequential peptide affinity*) [15] for detection by Western Blotting using anti-flag antibodies. A 426bp fragment corresponding to the 3' end of *mreBH* (devoid of its stop codon) was PCR-amplified using Phusion polymerase and primers AC938/933, and cloned into the plasmid pMUTINspa [16], using the *Hind*III and *Nco*I restriction sites. The resulting plasmid pAC616 was transformed into *B. subtilis* 168 and Campbell-like insertions into the *mreBH* locus were selected by resistance to erythromycin. The resulting strain ABS1324, was grown in the presence of IPTG to insure expression of the

gene downstream of *mreBH*. Chromosomal DNA of strain ABS1324 was subsequently transformed into strains NC103, 2521, 3725 and RCL78 (see Supplementary Table 5).

Luciferase assays

Luciferase experiments were carried out as previously described [17]. In brief, strains were first grown overnight in LB medium supplemented by 20 mM $MgSO_4$, then diluted in LB (20 mM $MgSO_4$, 0.5% xylose), adjusting all the cultures to an OD_{600} of 0.01 and incubated at 37°C. For growth in poor S medium, an extra growth/dilution step (in S supplemented with 20mM $MgSO_4$) was added to ensure adaptation and perfectly exponential growth of the cells. When cells reached OD_{600} of ~0.3, pre-cultures were diluted in the same medium to an OD_{600} of 0.005, and 200 µl of the cell suspension were distributed in a 96-well black plate (PerkinElmer) containing 10 µl of luciferin per well (luciferin final concentration, 1.5 mg ml^{-1}). The cultures were incubated at 37°C with agitation in a PerkinElmer Envision 2104 Multilabel Reader while luminescence and OD_{600} were recorded at 5 min intervals.

Western blot analysis

Cell extracts were prepared from cultures grown in LB (supplemented with 20mM $MgSO_4$, 1mM IPTG and 0.5% xylose) to an OD_{600} of 0.3-0.4. Bacteria were sedimented and resuspended in 50 mM Tris pH8, 50 mM glucose and 1mM EDTA supplemented with 1mg/ml lysozyme and a protease inhibitor cocktail at the concentration described by the supplier (Roche). Lysis buffer (50 mM Tris pH8, 500 mM NaCl, 1% nonidet P-40, 5mM $MgCl_2$, benzonase (Merck)) was then added. Following an incubation step of 20 min on ice, the samples were loaded into 10% SDS-PAGE gels. Proteins were transferred to polyvinylidene difluoride (PVDF) membranes (GE Healthcare) and used for western blot analysis. Blots were performed using polyclonal antibodies against MreB (1:10 000) or Mbl

(1:1 000) and monoclonal antibodies anti-flag M2 (Sigma) (1:10 000) to detect MreBH-SPA, in TBS-T (0.05% Tween) containing 5% milk. Anti-rabbit and anti-mouse-HRP conjugate (Sigma) were added at 1:10 000. Detection was performed with ECL detection kit (GE Healthcare).

Kymograph visualization of mobile and non-mobile patches

Kymograph analysis of MreB patches was performed as described previously [18] with a few modifications. Briefly, the following steps were carried out to directly visualize the mode of movement and the speed of mobile MreB patches. First, we cropped the regions containing individual cells within the full field of view and generated separate movies containing each single cell. Second, lines were drawn manually to mark the cell midline from pole to pole at the single cell level. Third, a series of lines perpendicular to the cell midline were generated automatically with a spacing of 2 pixels apart. Last, kymographs were examined based on the series of perpendicular lines at different positions of the midline. Time bars are all vertical with an interval of 1 s.

Automatic classification of patch movement based on mean-squared displacement (MSD) analysis

Automatic classification of patch movement as directed motion, random diffusion or constrained diffusion requires MSD analysis. Random walks and directed movements (in 2D) were simulated in MATLAB using parameters identical to those used in our experimental conditions (lag time=1s, trace duration = 15s). First, directed trajectories were generated ($N = 1\,000$, speed $v = 50$ nm s^{-1}) (typical traces are shown in Supplementary Fig. 8a). These simulated traces were analyzed by MSD (individual and average curves are plotted in Supplementary Fig. 8b-c). Then these MSD curves were fitted using classic quadratic model

used for directed motion ($MSD(t) = (vt)^2$) and classified as directed if their goodness-of-fit $R^2_{dir} \geq 0.8$. Based on this criterion, 99% of simulated trajectories were classified as directed and patch speed were correctly estimated from MSD curve ($v_{est} = 51.2$ nm s^{-1}, left panel in Supplementary Fig. 8g). Second, we generated random walks ($N = 1\,000$, diffusion coefficient $D = 0.01$ μm^2 s^{-1}). Typical traces are shown in Supplementary Fig. 8d and the resulting individual and average MSD curves are plotted respectively in Supplementary Fig. 8e-f. MSD curves were fitted using a linear model ($MSD(t) = 4Dt$) and classified as diffusing if $R^2_{diff} \geq 0.8$. According to this rule, around 60% of traces were correctly classified as diffused movement with an accurate estimation of diffusion coefficient $D_{est} = 0.010$ μm^2 s^{-1} (right panel in Supplementary Fig. 8g). Unsupervised classification of constrained patches was defined using time-lapse TIRFM acquisitions of fixed *B. subtilis* cells expressing GFP-MreB (RCL238). Cells were fixed with 4% formaldehyde in PBS (50:50, vol:vol) at room temperature and then washed twice in PBS. Patches were tracked along image sequences and the average MSD curves were calculated (Supplementary Fig. 8h) and appeared flat as compared to averaged MSD curves obtained with directed motion and Brownian diffusion. We concluded that constrained patches could be easily separated from mobile patches considering the maximal value of the MSD curve ($MSD_{constrained} < 0.05$ μm^2 s^{-1}).

In summary, tracked patches were classified without any supervision based on the value of the MSD curve and its fitting (Supplementary Fig. 8i). Patches with MSD maximum < 0.05 μm^2 s^{-1} were classified as constrained. Otherwise, the behavior of patch movement was classified as directed motion if $R^2_{dir} \geq 0.8$ and $R^2_{dir} > R^2_{diff}$, as random diffusion if $R^2_{diff} \geq 0.8$ and $R^2_{diff} > R^2_{dir}$ and unclassified if $R^2_{dir} < 0.8$ and $R^2_{diff} < 0.8$.

Analysis of MreB trajectories using cumulative distribution functions (CDFs)

Cumulative distribution functions (CDFs) of displacements is an alternative standard approach to obtain apparent diffusion coefficients by fitting them to an exponential function corresponding to 2D Brownian motion [19,20]. Using trajectories obtained with u-track and classified as *diffusive* by MSD analysis, frame-to-frame displacements (r) were calculated and pooled together for each movie. The CDFs of the displacement magnitudes was fitted using an analytical function that takes into account a two-state model:

$$P(r, \Delta t) = 1 - w_1 \exp(-\frac{r^2}{4D_1 \Delta t}) - (1 - w_1) \exp(-\frac{r^2}{4D_2 \Delta t})$$

where $P(r, \Delta t)$ is the cumulative probability of a displacement of magnitude r given the observation period Δt=1s, diffusion coefficients are D_1 and D_2, and the relative fraction between the two states is w_1. Finally, the apparent diffusion coefficients measured for each cell were averaged. All calculations were performed on MATLAB (Mathworks, R2014b), using a nonlinear least-square algorithm.

Determination of the minimum width and the average distance between patches

In our analysis based on u-Track, comet detection algorithm used for patch identifications could not provide measurements of positioning errors and patch widths, mostly because this method relies on intensity thresholding and watershed. To circumvent this, patches detected using u-track were individually fitted to measure their lateral dimensions and to estimate the accuracy of centroid localization using a 2D anisotropic Gaussian:

$$f(x, y) = B_0 + I_0 \exp\left[-\left(\frac{(x - x_0)\cos\alpha + (y - y_0)\sin\alpha}{\sigma_1}\right)^2 - \left(\frac{-(x - x_0)\sin\alpha + (y - y_0)\cos\alpha}{\sigma_2}\right)^2\right]$$

where B_0 and I_0 are the background intensity and amplitude of the Gaussian centered at (x_0, y_0), with lateral widths (σ_1 and σ_2), and α is the orientation of the Gaussian. Applying

this fitting model on our experiments provided approximated estimations of lateral dimensions of patch and the positioning accuracy.

To determine the spacing between patches, the distance between the centroids of closest neighboring patches was measured (N > 1 000 per condition).

Measurement of cell dimensions (for Supplementary Tables 1 and 2)

Phase contrast images were acquired on exponentially growing cells (OD_{600} ~ 0.3). Intensity profiles were obtained from lines manually drawn across the long and short axis of cells. Drastic intensity changes appeared where lines crossed the cell contour. The median value of the maximum and minimum intensity was used to produce two intersecting points. The distance between these two points yields cell length and width (Supplementary Fig. 12). All image processing was performed using MATLAB (Mathworks, R2014b).

Image acquisition and processing for microfluidics experiments

In order to follow *B. subtilis* and *E. coli* cells during media switch, we used a microfluidics flow chamber (CellASIC, EMD Millipore) and B04A microfluidics plates. *B. subtilis* cells carrying GFP-Mbl and *E. coli* cells carrying MreB-msfGFP^{SW} were first grown in S medium in shaking plates into early exponential phase (OD_{600} ~ 0.1), introduced into microfluidics plates filled with S medium, and then allowed to adapt for 60 minutes before LB was injected to replace S medium. Cell loading was achieved under pressure (5 psi) for 6 seconds, and constant media flow was maintained at 0.5 psi, which corresponds to roughly a flow rate of 10 µl h^{-1}. All experiments were carried out at 37 °C. Bright field (snapshot) and TIRFM movies (100 ms exposure every second for 1 minute) were acquired 5 minutes before, right after (T=0’) and every 5 minutes after the nutrient upshift. All TIRFM/SPT analyses were performed as described for other acquisitions. To calculate cell parameters (width, length,

area and growth rate), we first obtained binary images excluding background noises, by applying background subtraction (ball radius = 50 pixels = 3.2 µm) and intensity threshold (based on Otsu's method) (Supplementary Fig. 13a). Next, cells were individualized using watershed segmentation while objects others than bacterial cells, such as square pillars in CellASIC bacterial plates, were removed manually. Cell dimensions (length and width) were measured by an ellipse fitting. Cell area was calculated as the number of pixels within the cell contour multiplied by pixel area (Supplementary Fig. 13a). Single-cell growth rates were then calculated using the cell area. At the single cell level, time variation of volume $V(t)$ can be characterized by its growth rate $\mu(t)$ according to the relation $dV/dt = \mu(t)\, V(t)$. Volume of rod-shaped bacteria can be approximated by a cylinder $V(t) = (\pi/4)D^2(t)L(t)$, where $D(t)$ and $L(t)$ are the diameter and the length of the bacterium at time t. Then, the growth rate can be derived from cell dimensions with:

$$\mu(t) = \frac{2L(t)\frac{dD(t)}{dt} + D(t)\frac{dL(t)}{dt}}{D(t)L(t)}$$

or in a equivalent formulation:

$$\mu(t) = \frac{2A(t)\frac{dD(t)}{dt} + D^2(t)\frac{dL(t)}{dt}}{D(t)A(t)}$$

where $A(t)$ is the surface section of bacterium ($A(t) = D(t)L(t)$) at time t. In the case of *B. subtilis*, cell diameter is constant during nutrient shift and growth rate expression becomes simpler: $\mu(t) = (dA(t)/dt)/A(t) \approx (A_{i+1} - A_i)/(A_i \times dt)$, where A_i and A_{i+1} are cell areas in two adjacent frames at time t_i and t_{i+1} respectively, and $dt = t_{i+1} - t_i$ is the time interval in between.

TIRF imaging acquisition parameters and SPT analysis were as for cells under steady-state growth. Image processing was done using Fiji [21].

Bootstrap analysis

The bootstrap allows to create many new sets of data from the original dataset by sampling (with replacement) and leads to an empirical distribution of possible mean values, allowing to estimate a confidence interval [22,23]. Bootstrap samples were generated as follows: (i) all tracks (including the times when they were detected in the movie and the classification of their movement according the MSD analysis) and speeds of directed patches were exported for each processed movie, (ii) then new sets of data (N_{BS}=1 000) were generated by sampling the original dataset (the size of the original dataset and each bootstrap were kept identical), (iii) Classification of patch motion (directed, diffused, constrained and unclassified) and average speed of directed patches were estimated for each bootstrap sample as described in Methods, (iv) N_{BS} average values of mean speed and distribution percentage, standard errors and 95% confidence intervals were calculated (Supplementary Table 3). All calculations were performed on MATLAB (Mathworks, R2014b).

Statistical analysis

All statistical tests were performed using GraphPad Prism 6.05 and a non-parametric statistical test (two-tailed Mann-Whitney test with an alpha level of 5%).

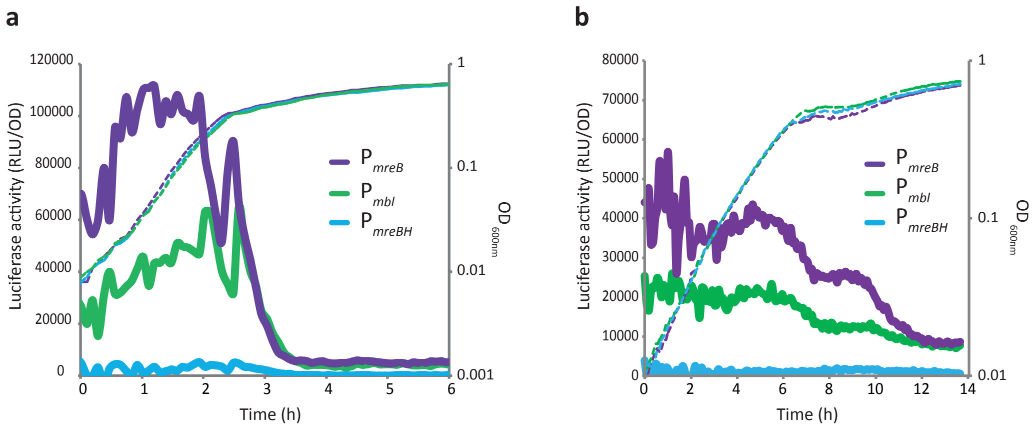

Supplementary Figure 1. Gene expression profile of *mreB, mbl* and *mreBH* during growth in LB (a) and S (b) media. Transcriptional fusions between *luc* and the full promoters of *mreB* (strain NC84, purple), *mbl* (NC87, green) or *mreBH* (NC89, blue) were integrated at the ectopic *amyE* locus. A typical experiment is presented where luminescence (Relative Luminescence Unit, RLU) corrected by the optical density at 600 nm (RLU/OD_{600}) is plotted as a function of time. The corresponding growth curves are shown as dotted lines.

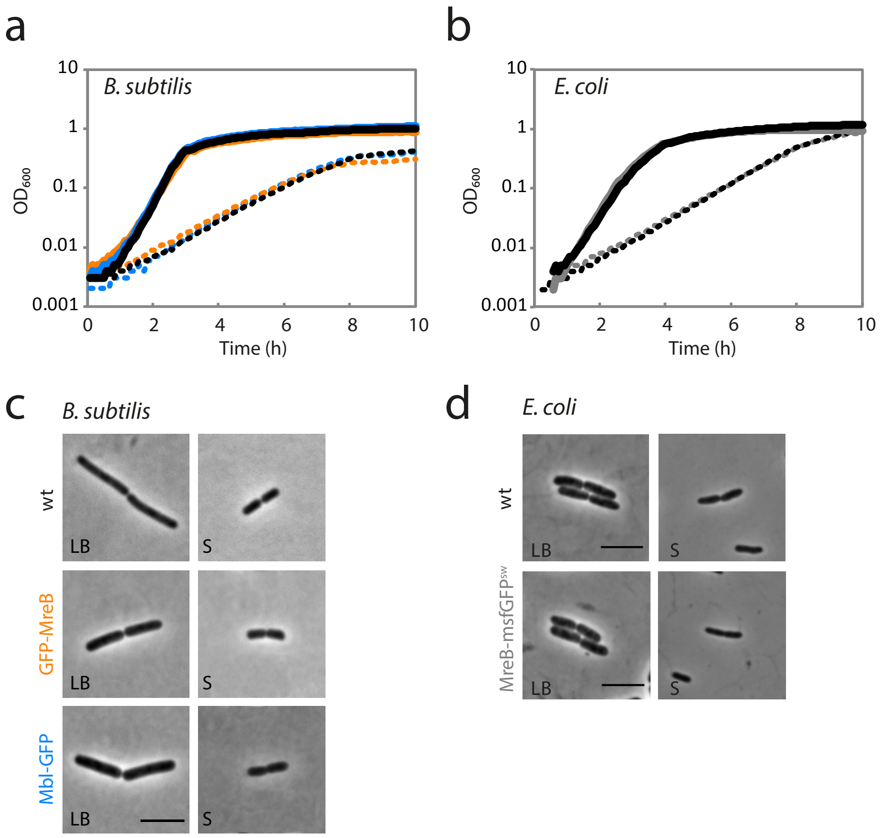

Supplementary Figure 2. Growth and morphology of strains carrying natively expressed fluorescent protein fusions to MreB proteins. (a,b) Growth curves (OD_{600}) of *B. subtilis* cells natively expressing GFP-MreB (strain NC103, orange) and Mbl-GFP (2521, blue) (a), and of *E. coli* cells expressing MreB-msfGFP^{SW} (N050, grey) (b) grown in LB (solid line) and S (dotted line) media at 37°C**.** Strains carrying the *gfp* fusions grow at the same rate than their respective wild-type control strain (black) in both media. **(c, d)** Phase contrast images of exponentially growing *B. subtilis* wild-type cells (wt, 168), cells carrying GFP-MreB (NC103) or Mbl-GFP (2521) (c), and *E. coli* wild-type cells and cells carrying MreB-msfGFP^{SW} (d) grown in LB and S media, as indicated, at 37 °C**.** Scale bars: 5 µm.

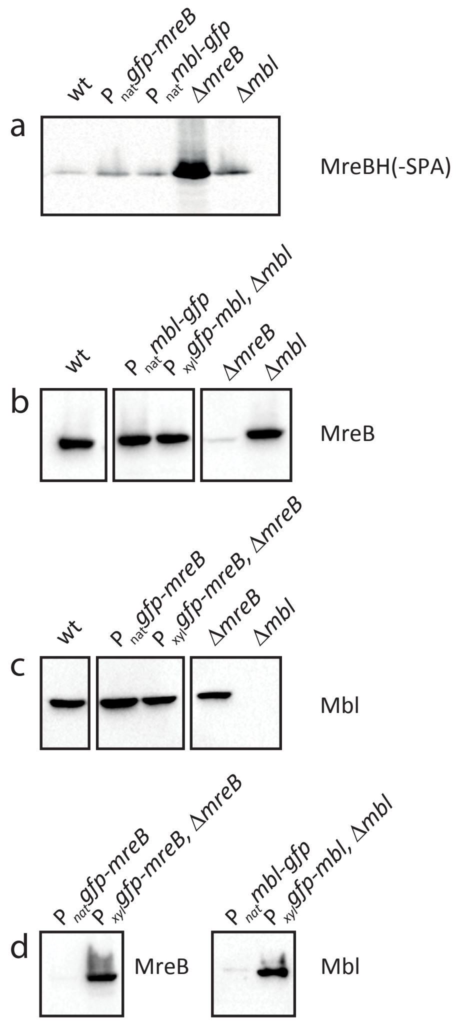

Supplementary Figure 3. **Immunoblot analysis of MreBH (a), MreB (b) and Mbl (c) protein levels in *B. subtilis* cells expressing *gfp* fusions to *mbl* or *mreB* under native or inducible promoters**. Samples were taken from exponentially growing cultures of wild-type cells (wt, strain 168), mutant cells for *mreB* (Δ*mreB*, 3725) or *mbl* (Δ*mbl*, RCL78), and of cells expressing translational fusion between *gfp* and either *mreB* or *mbl* as only copy of the corresponding *mreB*-like gene in the genome, under control of the native promoter (P_{nat}) or of a xylose-inducible promoter (P_{xyl}), as follows. **(a)** Comparison of the levels of MreBH (as MreBH-SPA fusion expressed from the native *mreBH* locus) in cells expressing *gfp* fusions to *mreB* (P_{nat} *gfp-mreB*; CcBs121) or to *mbl* (P_{nat} *mbl-gfp*; CcBs125) under control of their respective native promoters. **(b)** Comparison of the levels of MreB in cells expressing *gfp* fusions to *mbl* under control of its native (P_{nat} *mbl-gfp*; 2521) or a xylose-inducible (P_{xyl} *gfp-mbl* in Δ*mbl*; 2523) promoter. **(c)** Comparison of the levels of Mbl in cells expressing *gfp* fusions to *mreB* under control of its native (P_{nat} *gfp-mreB*; NC103) or a xylose-inducible (P_{xyl} *gfp-mreB* in Δ*mreB*; RCL238) promoter. **(d)** Comparison of the levels of natively expressed and xylose-inducible GFP fusions to MreB (NC103, RCL238 respectively) and to Mbl (2521, 2523 respectively). Raw immunoblots are presented as Supplementary Fig. 16.

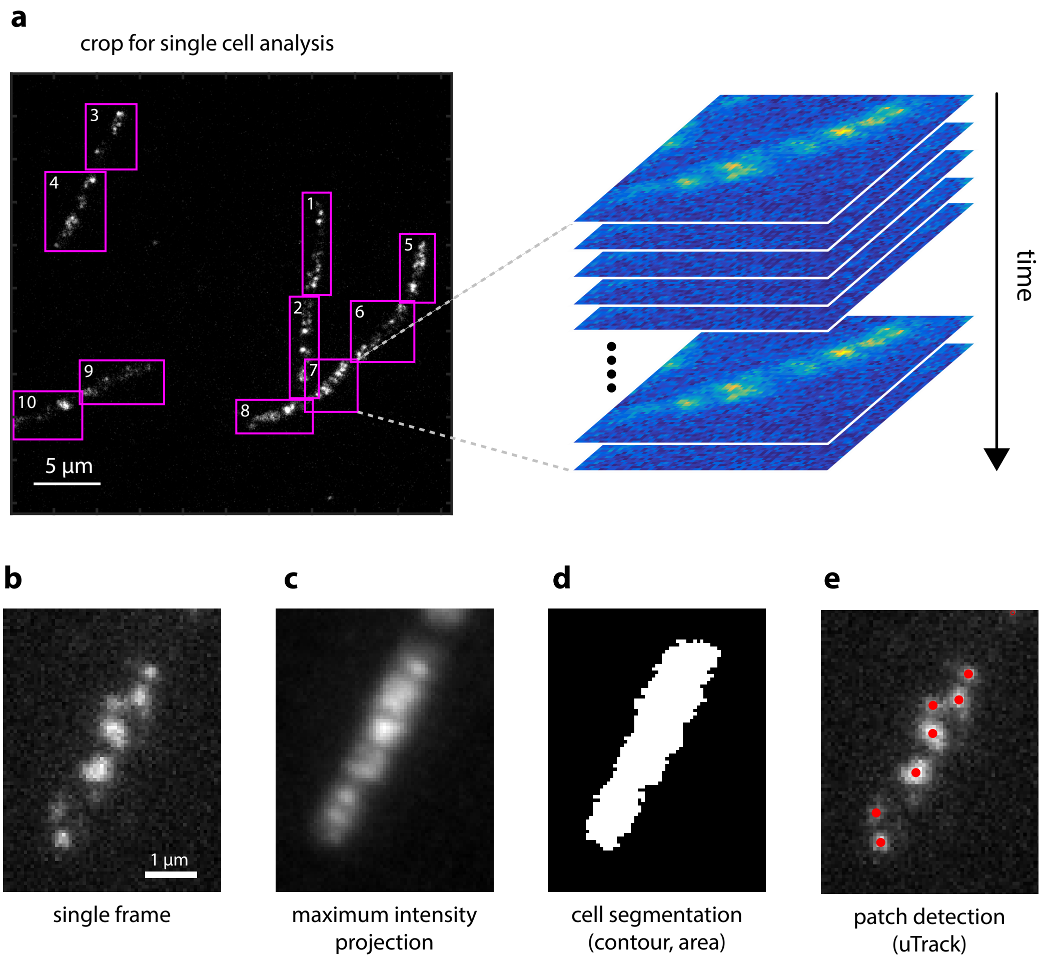

Supplementary Figure 4. **Flow-chart of MreB patch identification in a representative *B. subtilis* cell**. **(a)** Image analysis at the single cell level. Raw TIRFM time-lapse images of single cells were cropped out (magenta areas) of the full field of view to produce single cell movies. **(b)** Single frame from a time-lapse movie of a *B. subtilis* cell. **(c)** Maximum intensity projection of all frames in the movie. **(d)** Cell contour segmentation based on the maximum intensity projection. **(e)** Identification of the centroids of MreB patches using the u-track and MATLAB softwares.

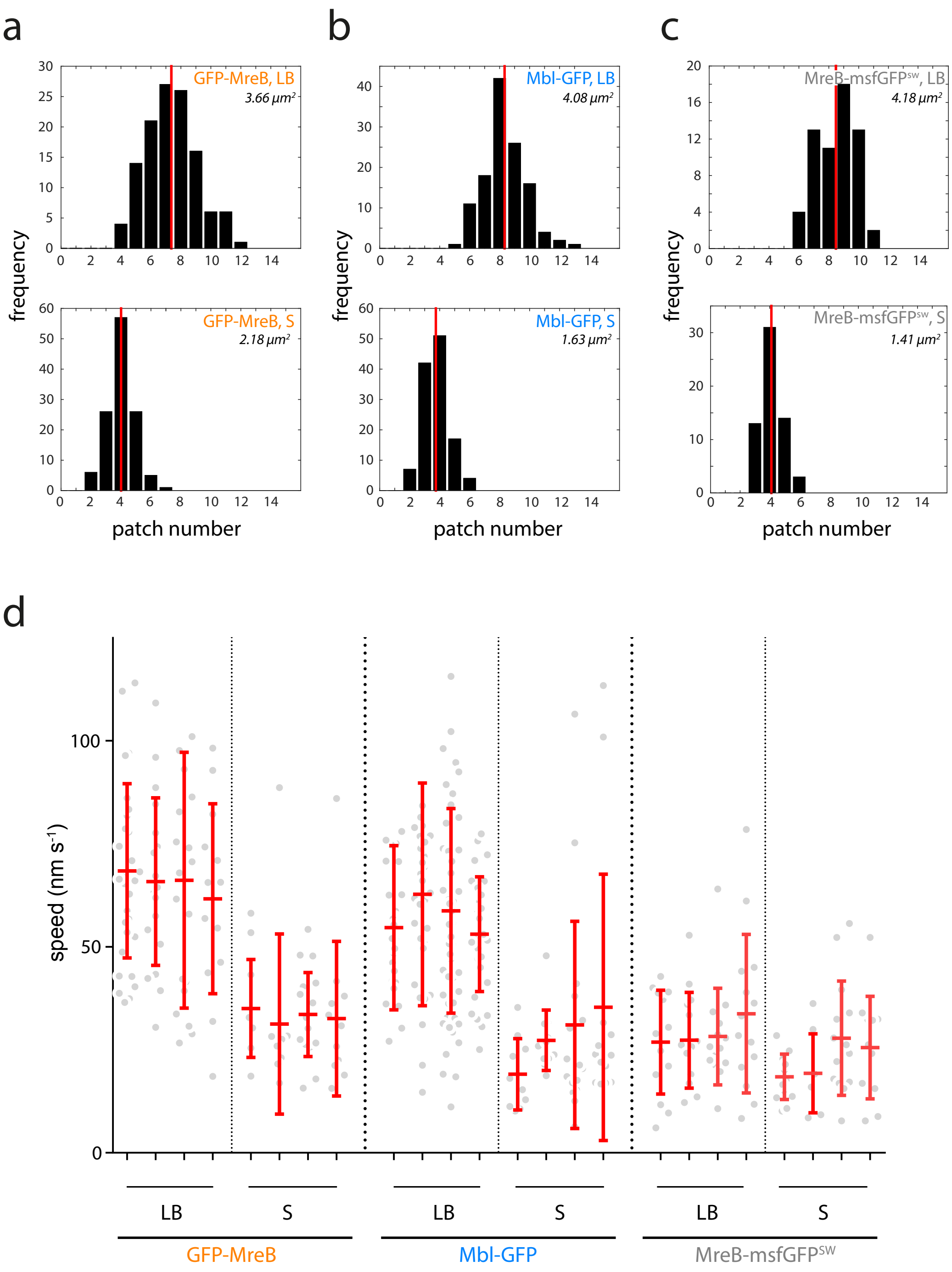

Supplementary Figure 5. MreB patch number and speed in individual cells. (a-c) Histograms of patch number per TIRFM frame in representative *B. subtilis* single cells carrying natively expressed GFP-MreB (a) and Mbl-GFP (b), and in *E. coli* cells carrying MreB-msfGFP^{SW} (c) grown in LB (top panels) and S (bottom panels) media. Patch number per cell is averaged over the entire movie (red solid line). Cell area under TIRF illumination is indicated in each panel (top right corner). (**d**) Scatter plot of the speed of directed patches obtained from individual tracks in single cells. 4 different cells are represented per fusion and condition. Averaged speeds are shown in red; error bars correspond to the standard deviation.

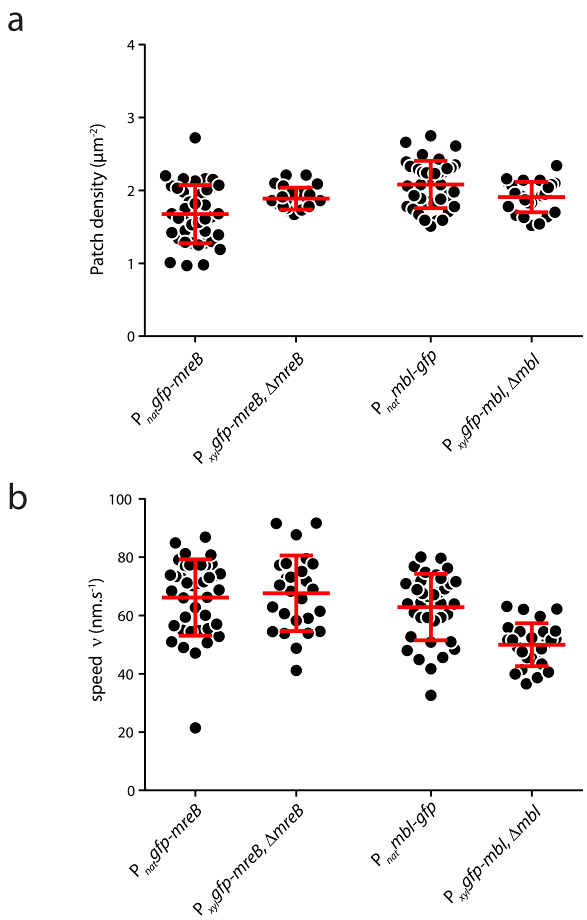

Supplementary Figure 6. **Patch density and speed distributions of natively expressed and inducible GFP fusions to MreB and Mbl in *B. subtilis*.** Scatter plots of the distribution of total patch density **(a)** and directed patch speed (ν) **(b)** in exponentially growing cells expressing GFP-MreB and Mbl-GFP fusions under control of their native (P_{nat} *gfp-mreB*, NC103, n = 39; P_{nat} *mbl-gfp*, 2521, n = 37) or a xylose-inducible (P_{xyl} *gfp-mreB* in Δ*mreB*, n = 26, RCL238; P_{xyl} *gfp-mbl* in Δ*mbl*, 2523, n = 26) promoter. Cells were grown at 37°C in LB medium. Plotted averages and standard deviations are shown in red. Data are a compilation of at least 2 independent experiments.

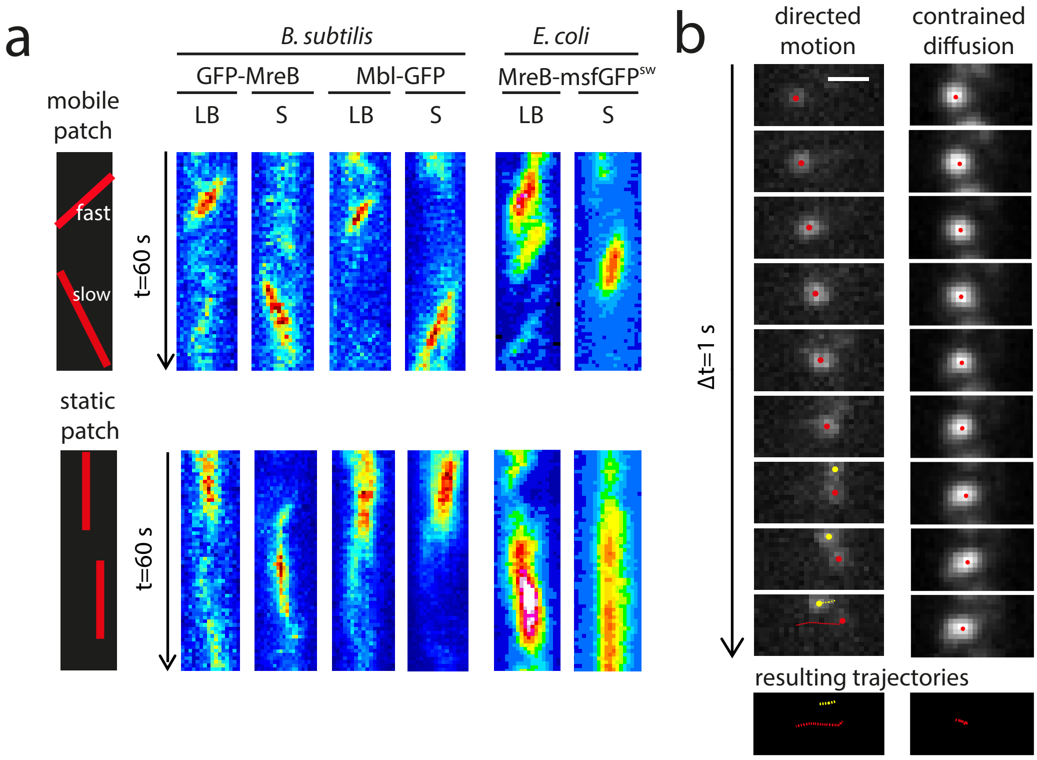

Supplementary Figure 7. Not all MreB patches exhibit circumferential, directed movement. **(a)** Representative kymographs showing motile (top panels) and static (bottom panels) patches in *B. subtilis* cells carrying native GFP-MreB and Mbl-GFP fusions, and in *E. coli* cells carrying MreB-msfGFP^{SW} grown at 37 °C in LB and S media as indicated. Pixel size: 64 nm. Partially diffusive behavior is also visible in the kymographs (short traces or background signal) **(b)** Time-lapse images of two subpopulations of GFP-MreB patches in *B. subtilis* cells grown in LB: directed motion (left) and constrained diffusion (right). The SPT algorithm employed is able to separate two adjacent trajectories (red and yellow). Positions of the patches are summed up to show the trajectories on the bottom panels. Pixel size: 64 nm. Time interval: 1 s. Scale bar: 0.5 µm.

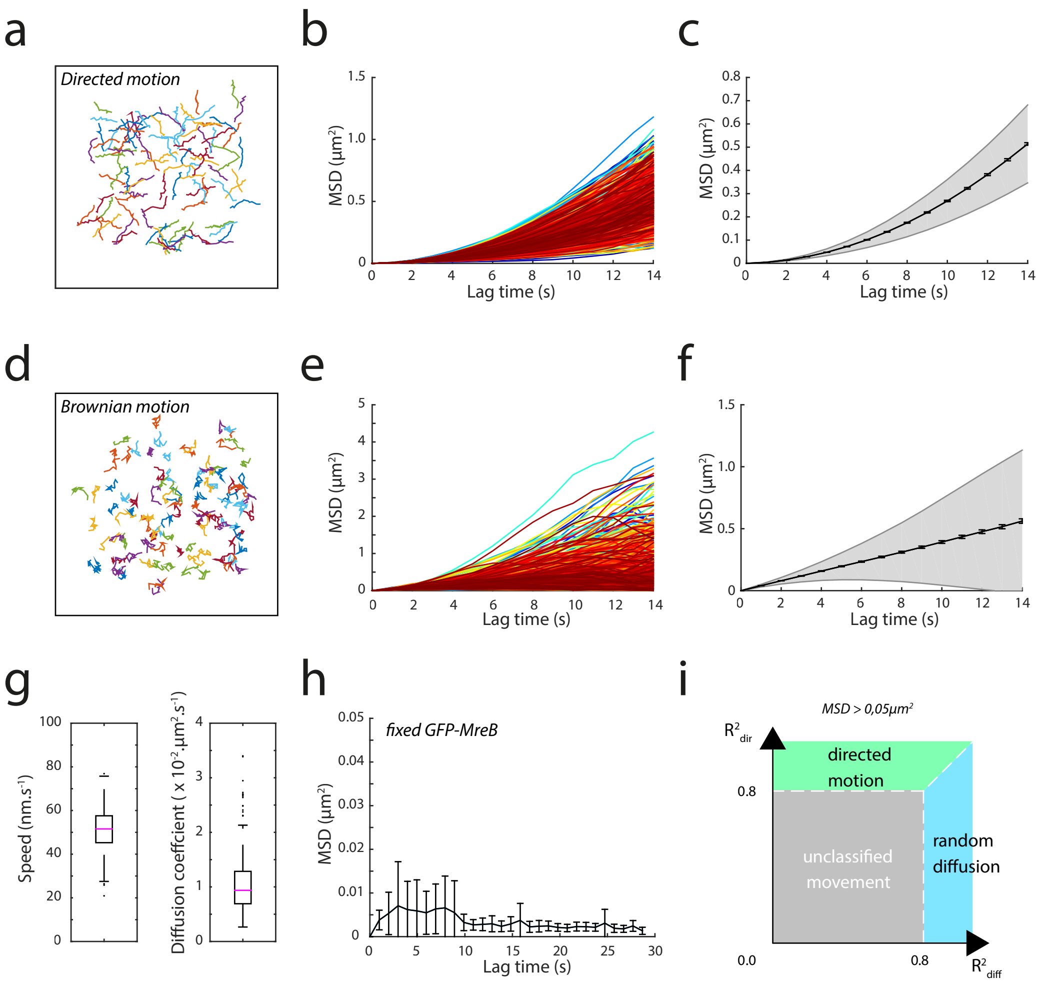

Supplementary Figure 8. Automatic classification of patch movement based on mean-squared displacement (MSD) analysis. Simulations of directed and Brownian motions (N=1000 tracks) were analyzed by MSD to identify robust criteria allowing to classify them as directed motion (a-c) or random diffusion (d-f). **(a, d)** Representative trajectories (only 100 traces were plotted for clarity). Individually plotted **(b, e)** and averaged **(c, f)** MSD curves. The grey areas represent the weighted standard deviation over all MSD curves. **(g)** Boxplot distributions of patch speed and diffusion coefficient estimated from MSD fitting of trajectories automatically classified as directed or random, respectively. **(h)** MSD curve of immobilized MreB patches. TIRFM time-lapse acquisitions were performed on fixed *B. subtilis* cells carrying the *gfp-mreB* fusion under control of a xylose-induced promoter (P_{xyl} *gfp-mreB* Δ*mreB*, strain RCL238, $n = 2$). Patches were tracked using u-track. Error bars depict the weighted standard deviation. **(i)** Schematic representation of our automatic classification of movements based on MSD fitting analysis. MSD curves with: a maximal value <0.05 µm^2 are classified as constrained patches, a maximal value >0.05 µm^2 are classified as directed motions if the fitting of the MSD curve with a quadratic model ($MSD_{dir}(t) = (vt)^2$) provides a goodness-of-fit $R^2_{dir} \geq 0.8$ and $R^2_{dir} > R^2_{diff}$ (green area), or as random motion if the fitting of the MSD curve with a linear model ($MSD_{diff}(t) = 4Dt$) provides a goodness-of-fit $R^2_{diff} \geq 0.8$ and $R^2_{diff} > R^2_{dir}$ (blue area), or unclassified otherwise (grey area).

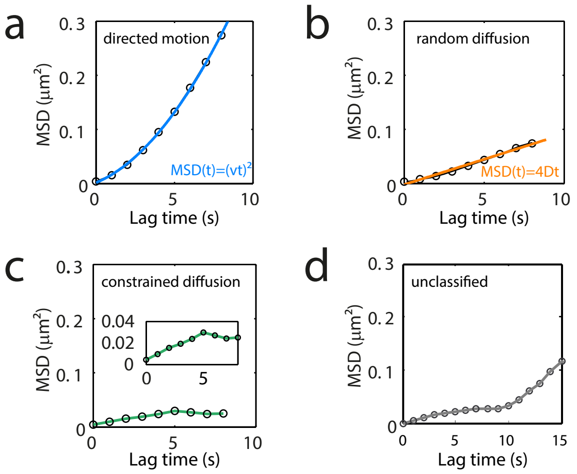

Supplementary Figure 9. MSD curves of representative MreB patches. Patches undergoing active, directed motion (a) and random diffusion (b) can be fitted to second and first degree polynomials, respectively. Constrained patches (c) reach a low, saturating MSD value (inset presents the same example with expanded Y axis). Some MSD curves exhibit mixed characteristics preventing the automatic classification, and thus were assigned to the unclassified category (d).

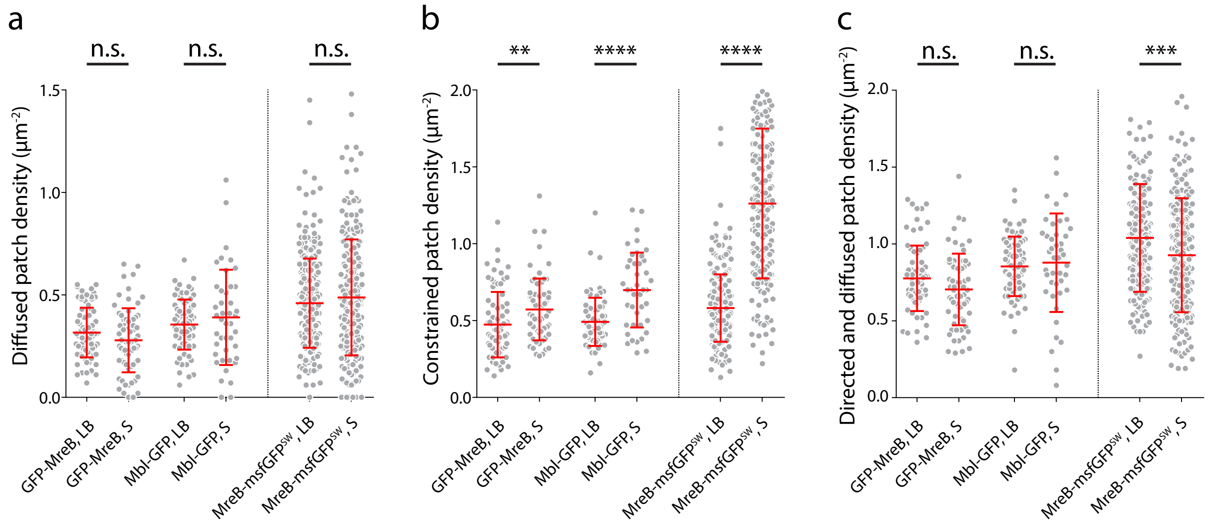

Supplementary Figure 10. Density of MreB patch sub-populations. Analyses of TIRFM acquisitions performed on *B. subtilis* cells carrying GFP-MreB and Mbl-GFP, and in *E. coli* cells carrying MreB-msfGFP^{SW} grown in rich (LB, respectively $n = 77$, $n = 77$, $n = 276$) and poor (S, respectively $n = 69$, $n = 43$, $n = 239$) media. Density of MreB patches classified as diffused (a), constrained (b) and the sum of directed and diffused (c). The density of diffused patches is not significantly (n.s.) different between rich and poor media for all three fluorescent fusions. However, the density of constrained patches is highly increased in poor medium relative to rich medium in *E. coli*. Note that the density of constrained patches also significantly (but to a much lesser extent) increases in poor medium in *B. subtilis*. This weak variation is likely to result from a better assignment of 'unclassified' patches due to slower motion at a lower growth rate. In red are plotted averages and standard deviations. Data are a compilation of at least 2 independent experiments. Plotted averages and standard deviations are shown in red. Distributions in LB and S media were compared using the Mann-Whitney non parametric statistical test (****, p<0.0001; ***, 0.0001<p<0.001; **, 0.001<p<0.01; *, 0.01<p<0.05; n.s., p>0.05).

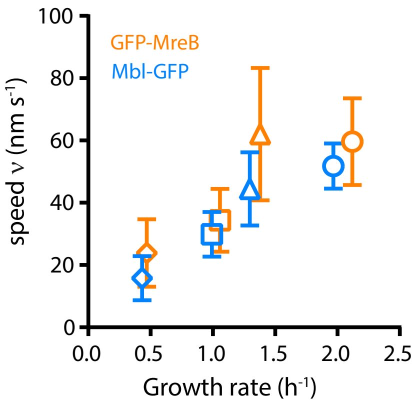

Supplementary Figure 11. Speed of MreB and Mbl circumferentially moving patches in different growth media. To achieve various growth rates, cells were grown in increasingly rich media: M9SE (diamonds; MreB, $n = 25$; Mbl, $n = 53$), S (squares; MreB, $n = 69$; Mbl, $n = 43$), CH (triangles; MreB, $n = 18$; Mbl, $n = 22$) and LB (circles; MreB, $n = 77$; Mbl, $n = 77$). Error bars correspond to the standard deviation. Data are a compilation of at least 2 independent experiments.

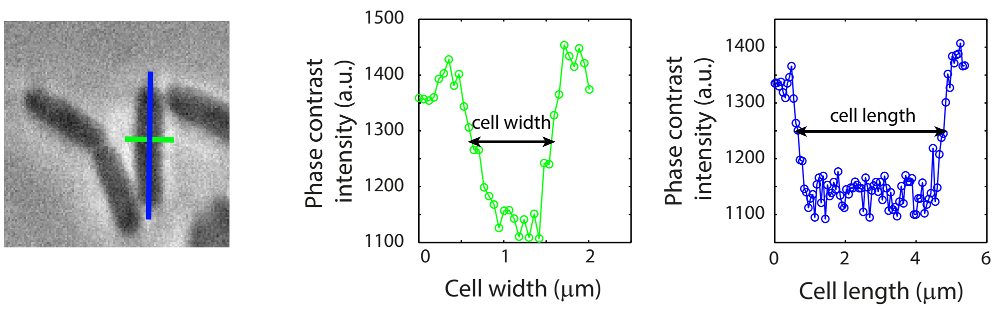

Supplementary Figure 12. Measurement of cell dimensions during steady state growth. Cell length and width measurement of a representative *E. coli* cell. Full width at half maximum (FWHM) of the phase-contrast intensity profile line was used to calculate cell width and length, respectively.

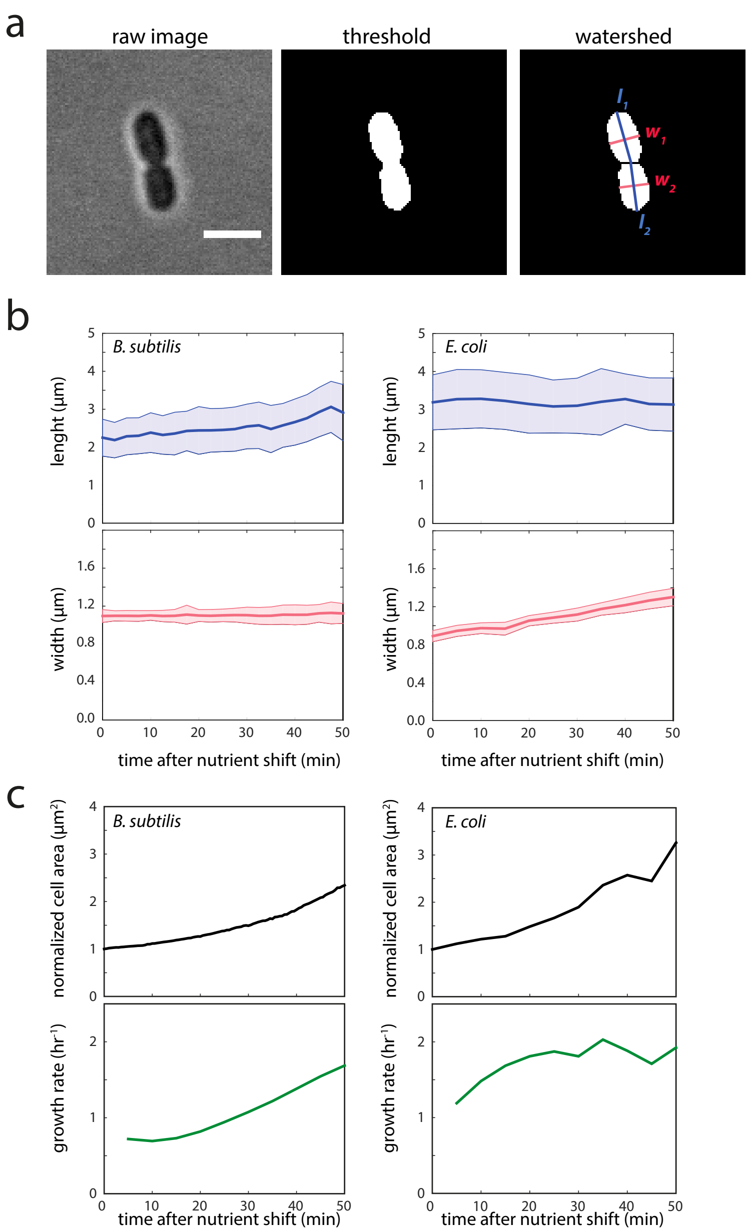

Supplementary Figure 13. Adaptative response in *B. subtilis* (expressing native GFP-MreB or Mbl-GFP fusions) and in *E. coli* (expressing native MreB-msfGFP^{SW}) upon nutrient upshift in a perfusion-based microfluidic device. (a) Image processing flowchart for the analysis of cell dimension. From raw phase contrast images (left), a background subtraction and intensity threshold value was applied to each 2D image to transform the grayscale image into a binary one (middle). Cells were individualized by watershed segmentation (right) and cell length (*l*) and width (*w*) were automatically measured. Scale bar: 2 µm. **(b, c)** Cell dimensions (cell length and cell width) (b), cell area and growth rate (c) during nutrient upshift, from S medium to LB medium. Time is in minutes. Growth rates (green) were measured from cell area (see Methods). At least 36 cells were measured (*B. subtilis*, $n = 90 - 111$; *E. coli*, $n = 36 - 82$). Data are a compilation of at least 2 independent experiments. Solid lines indicate average values; shaded areas indicate standard deviation.

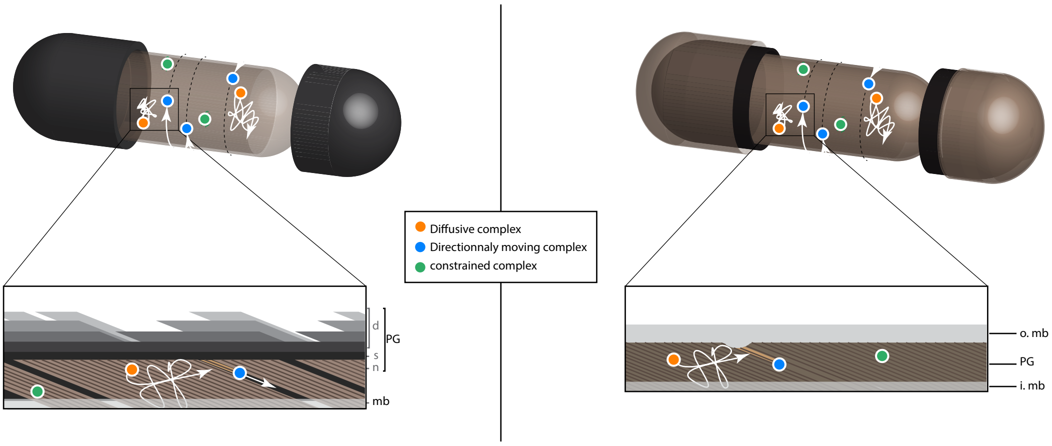

Supplementary Figure 14. Models of PGEM-mediated insertion of PG bands in bacteria. Schematic illustrations of the PGEMs in different physiological states in Gram-positive (left) and Gram-negative (right) bacteria. Zoomed-in are views from inside the cells, but cytoplasmic membranes have been partially removed for clarity. In the Gram-positive bacterium, an activated complex (blue dot) inserts new PG strands below the stress-bearing PG layer. In the Gram-negative bacterium, a deformation of the membrane drives the localization of the active PGEM that inserts new PG bands between existing strands in a "3-for-1" mechanism (see Supplementary Fig. 15).

PG, Peptidoglycan; d, discontinuous PG layers; s, stress-bearing PG layer; n, new uncompleted innermost PG layer; o. mb, outer membrane.

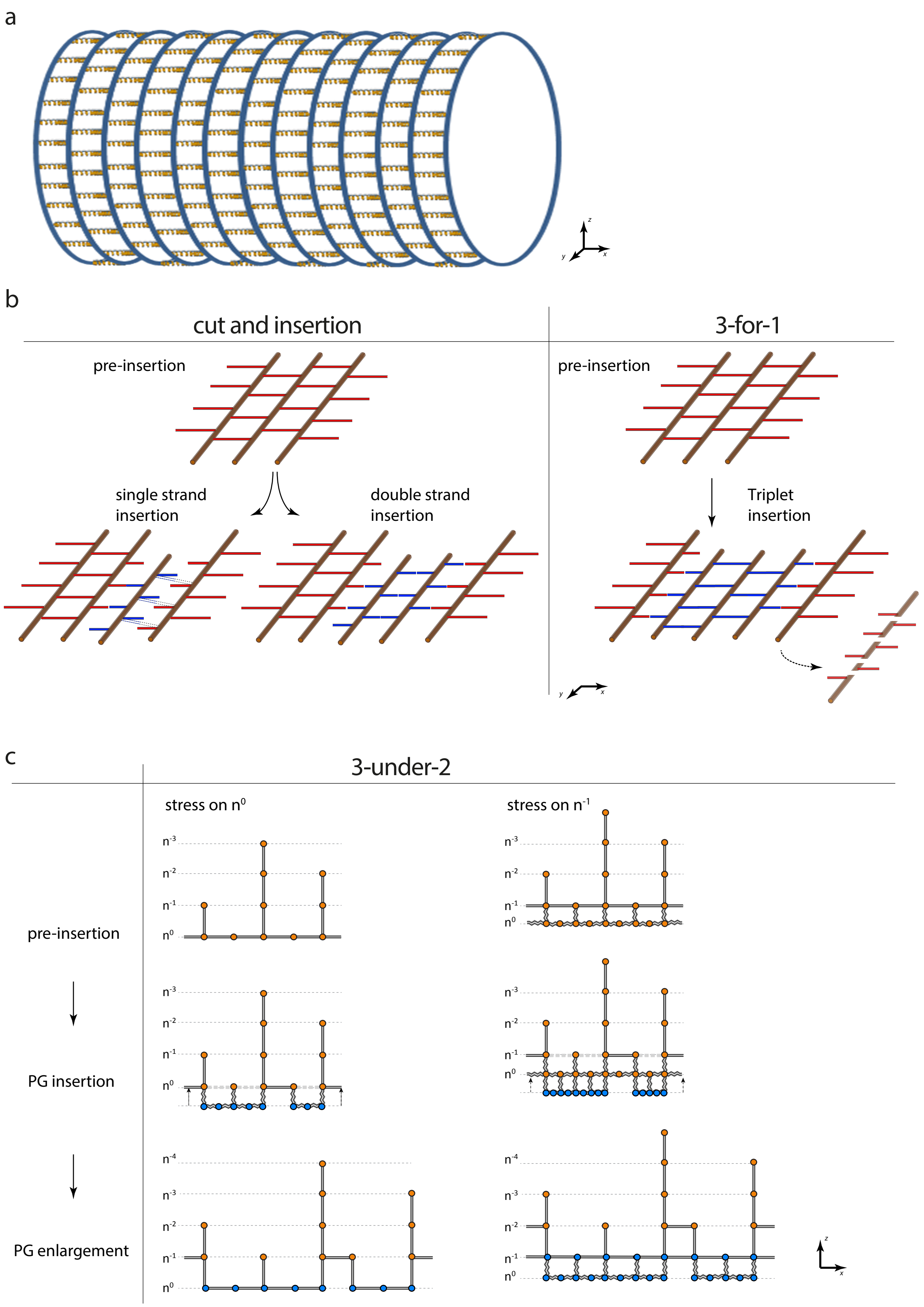
a
b
cut and insertion
3-for-1
pre-insertion
pre-insertion
single strand
insertion
double strand
insertion
Triplet
insertion
c
3-under-2
stress on n⁰
stress on n⁻¹
pre-insertion
PG insertion
PG enlargement

Supplementary Figure 15. Mechanistic models of peptidoglycan synthesis in rod-shaped bacteria. **(a)** Idealized and simplified 3D representation of a peptidoglycan layer. Glycan strands (blue rings) run perpendicular to the long axis of the cell, bridged by cross-linked stem peptides (yellow springs). **(b)** Strategies of glycan strand insertion in a 2D (x, y) peptidoglycan layer. In a "cut-and-insertion" strategy (left), peptide bridges are cleaved, allowing new strands to be inserted. Odd numbers of strands (e.g. single strand insertion) impose a stress on the cross-links due to un-alignment (dotted lines) of the stem peptides, while even numbers (e.g. double strand insertion) do not. In the "3-for-1" insertion, a triplet of glycan strands is replacing an existing strand of the meshwork. The net increase in strand number being even, no stress is introduced. Each insertion involves the recycling of old material representing after one cell cycle (one doubling) 50% of the initial peptidoglycan mass, and requires producing 1.5-fold this initial mass. Red lines represent peptide bridges, brown tubes for GluNac-MurNac sugar chains. **(c)** "3-under-2" glycan strand insertion modes in a multilayered peptidoglycan meshwork in 2D side view (x, z). Two possibilities are explored in which stress either is borne by the innermost (newest) layer only (left), or by the penultimate (n^{-1}) layer (right). Note that the number of glycan chains inserted per fully extended crosslink is two-fold bigger when stress is borne on layer n^{-1}. Arbitrarily, only five layers of PG are presented (numbered n^0 to n^{-4} from the innermost to the outermost). It is assumed that bundles of 3 to 9 glycan strands are inserted. Peptide bridges are shown in gray, in a relaxed conformation (broken lines), in full extension (straight lines) or hydrolyzed (dashed lines). Circles indicate cross-sections of glycan chains newly (blue) or previously inserted (orange).

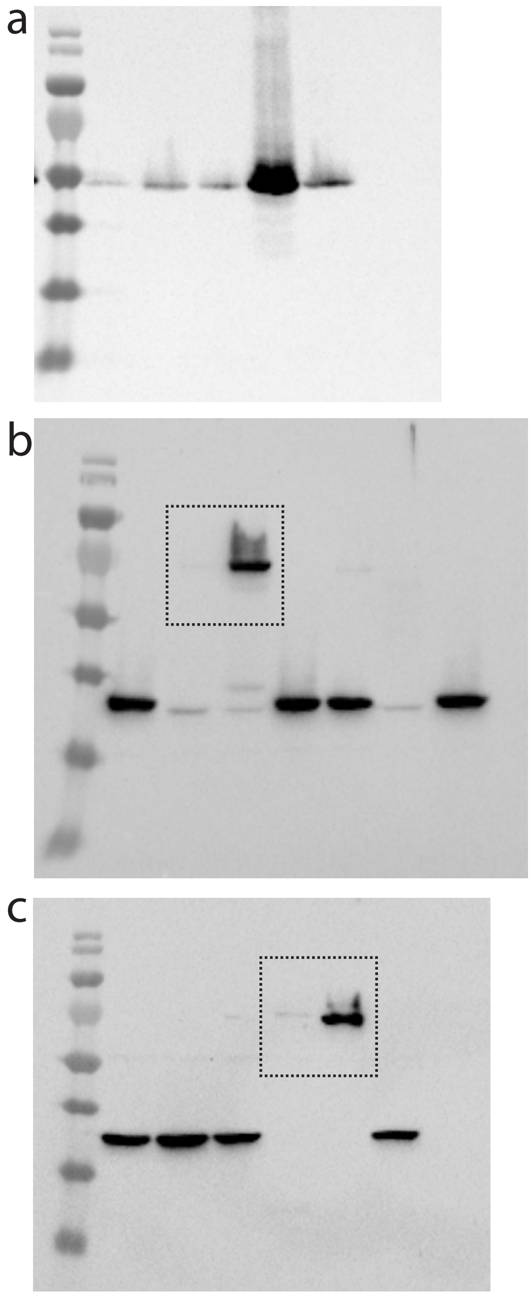

Supplementary Figure 16. **Raw immunoblot of *B. subtilis* cells extract expressing *gfp* fusions to *mbl* or *mreB* under native or inducible promoters, using anti-MreBH (a), anti-MreB (b) and anti-Mbl (c) antibodies**. Boxes indicate cropped areas used for panel d of Supplementary Figure 3.

Supplementary Table 1. Growth rate and cell dimensions of wild-type strains

	B. subtilis, WT				*E. coli*, WT	
	LB	CH	S	M9SE	LB	S
**τ (min)**	25.2 ± 3.50	40.0 ± 5.50	58.6 ± 8.30	109.0 ± 10.1	26.2 ± 2.80	68.0 ± 8.70
**L (µm)**	5.63 ± 0.24	4.05 ± 0.18	2.81 ± 0.13	1.98 ± 0.10	5.6 ± 0.34	3.42 ± 0.17
**D (µm)**	0.81 ± 0.06	0.82 ± 0.07	0.83 ± 0.09	0.81 ± 0.07	1.08 ± 0.07	0.81 ± 0.04

*Generation time (τ) is the average ± SD of three independent experiments. Cell dimensions (L and D) are the average ± SD of at least 25 cells per strain and condition.

Supplementary Table 2. Growth rate and cell dimensions of *B. subtilis* and *E. coli* strains expressing native GFP fusions to MreB proteins

	B. subtilis								*E. coli*	
	GFP-MreB				Mbl-GFP				MreB-msfGFP^{sw}	
	LB	CH	S	M9SE	LB	CH	S	M9SE	LB	S
**τ (min)**	28.3±3.6	43.5±6.3	56.8±9.5	128±10.3	30.5±4.2	46.3±7.5	60.4±8.9	139±13.5	20.9±0.9	54.8±0.1
**L (µm)**	4.70±0.27	3.61±0.19	2.48±0.14	1.68±0.09	5.05±0.24	3.97±0.23	2.51±0.15	1.75±0.11	3.84±0.80	2.97±0.76
**D (µm)**	0.88±0.05	0.86±0.05	0.83±0.07	0.81±0.04	0.84±0.04	0.83±0.07	0.82±0.09	0.80±0.07	1.12±0.20	0.72±0.07

* Generation time (τ) is the average ± SD of three independent experiments. Cell dimensions (L and D) are the average ± SD of at least 25 cells per strain and condition.

Supplementary Table 3. Influence of the minimal number of steps used for tracking and MSD analysis

			**Step #**	**Traces #**	**Directed %**	**Diffused %**	**Constrain. %**	**Unclassif. %**	**Speed (*M±SD*)[a] (nm s^{-1})**	**D (*M±SD*)[a] ($x10^{-3}$ $µm^2$ s^{-1})**
B. subtilis	MreB	LB	4	4418	30,94	16,34	43,82	8,90	63,16 ± 12,39	3,25 ± 0,80
			8	2301	35,81	22,60	27,34	14,25	49,27 ± 10,99	2,96 ± 0,91
	MreB	S	4	2336	22,99	13,53	59,55	3,94	37,84 ± 15,76	2,25 ± 0,69
			8	1374	34,93	18,85	40,98	5,24	30,94 ± 6,25	1,81 ± 0,52
	Mbl	LB	4	5525	32,49	17,00	42,05	8,47	52,07 ± 6,75	2,75 ± 0,54
			8	3337	39,20	22,21	26,13	12,47	43,83 ± 5,09	2,48 ± 0,56
	Mbl	S	4	1422	22,93	13,22	59,21	4,64	31,81 ± 8,80	1,80 ± 0,79
			8	937	30,95	18,46	44,61	5,98	24,67 ± 4,30	1,54 ± 0,53
E. coli	MreB	LB	4	10803	27,79	14,79	49,20	8,22	30,66 ± 4,98	1,57 ± 0,47
			8	7765	35,56	19,69	33,59	11,17	27,77 ± 3,19	1,47 ± 0,41
	MreB	S	4	6924	18,11	12,51	65,25	4,13	27,20 ± 9,95	1,22 ± 0,59
			8	5106	22,39	15,75	56,78	5,09	21,77 ± 5,42	1,04 ± 0,49

Classification of movement behavior: directed, diffused, constrained, unclassified; D, diffusion coefficient of diffused patches.
[a] M, measured mean with standard deviation, SD

Supplementary Table 4. Directed patch density and speed, and percentage of patch sub-populations

Strains	Medium	Directed patch density			Directed patch speed		
		M±SD	*M*±SE*	*Conf. interval*	*M±SD*	*M*±SE*	*Conf. interval*
NC103	LB	0,46 ± 0,17	0,46 ± 0,01	0,43 - 0,49	59,6 ± 13,97	59,62 ± 0,91	57,92 - 61,45
	S	0,44 ± 0,22	0,43 ± 0,02	0,39 - 0,48	36,04 ± 17,34	36,36 ± 1,09	34,39 - 38,51
	CH	0,31 ± 0,08	0,31 ± 0,07	0,18 - 0,45	57,38 ± 1,47	57,43 ± 5,63	46,7 - 68,65
	M9SE	0,5 ± 0,26	0,48 ± 0,05	0,37 - 0,59	23,07 ± 12,4	23,29 ± 1,76	20,06 - 26,82
2521	LB	0,5 ± 0,14	0,5 ± 0,01	0,47 - 0,53	51,76 ± 7,27	51,74 ± 0,61	50,56 - 52,93
	S	0,49 ± 0,27	0,49 ± 0,03	0,43 - 0,55	31,04 ± 10,12	31,05 ± 1,38	28,5 - 34,09
	CH	0,28 ± 0,17	0,28 ± 0,02	0,24 - 0,32	47,72 ± 17,87	47,75 ± 3,38	41,26 - 54,26
	M9SE	0,36 ± 0,27	0,36 ± 0,03	0,3 - 0,43	16,34 ± 11,77	16,19 ± 1,22	13,76 - 18,34
RCL238	LB	0,38 ± 0,11	0,38 ± 0,02	0,35 - 0,42	67,65 ± 12,95	67,65 ± 1,2	65,38 - 69,9
2523	LB	0,32 ± 0,1	0,32 ± 0,02	0,29 - 0,36	49,96 ± 7,37	50,0 ± 1,22	47,72 - 52,39
RWSB18/GFP	LB	0,24 ± 0,2	0,24 ± 0,04	0,17 - 0,32	76,73 ± 24,97	77,93 ± 5,22	67,71 - 88,26
RWSB18/RFP	LB	0,22 ± 0,21	0,22 ± 0,03	0,16 - 0,29	106,58 ± 17,53	105,53 ± 5,5	93,96 - 115,54
ABS2408/GFP	LB	0,37 ± 0,18	0,37 ± 0,05	0,27 - 0,47	69,37 ± 27,63	69,82 ± 3,46	62,75 - 76,78
ABS2408/RFP	LB	0,3 ± 0,26	0,3 ± 0,04	0,22 - 0,39	80,39 ± 32,65	79,36 ± 5,62	67,72 - 90,2
NO50	LB	0,58 ± 0,29	0,58 ± 0,01	0,56 - 0,6	30,3 ± 5,43	30,32 ± 0,31	29,73 - 30,9
	S	0,44 ± 0,25	0,44 ± 0,01	0,41 - 0,47	26,02 ± 10,52	26,25 ± 0,66	25,03 - 27,51

Strains	Medium	% of directed patches			% of diffusing patches		
NC103	LB	30,24 ± 10,41	30,37 ± 0,79	28,84 - 32,01	21,4 ± 7,38	21,39 ± 0,77	19,84 - 22,94
	S	30,47 ± 13,87	30,54 ± 1,15	28,3 - 32,78	20,72 ± 11,19	20,39 ± 1,12	18,15 - 22,66
	CH	22,21 ± 5,42	22,19 ± 5,02	13,37 - 32,96	42,21 ± 9,99	42,25 ± 7,66	26,52 - 56,15
	M9SE	33,85 ± 17,87	34,35 ± 2,91	28,66 - 40,02	18,9 ± 18,25	18,42 ± 2,95	12,85 - 24,29
2521	LB	31,07 ± 7,86	31,12 ± 0,79	29,54 - 32,7	22,43 ± 7,24	22,38 ± 0,75	20,87 - 23,95
	S	27,19 ± 16,46	27,27 ± 1,39	24,53 - 30,04	21,87 ± 12,61	21,77 ± 1,57	18,66 - 24,77
	CH	20,48 ± 12,14	20,72 ± 1,48	17,86 - 23,71	26,2 ± 10,4	26,01 ± 2,1	21,69 - 29,89
	M9SE	27,49 ± 19,44	27,16 ± 1,87	23,59 - 31,13	16,62 ± 15,22	16,3 ± 1,9	12,55 - 19,93
RCL238	LB	22,83 ± 7,32	22,86 ± 0,94	21,08 - 24,72	22,93 ± 5,87	22,96 ± 1,14	20,74 - 25,21
2523	LB	18,63 ± 5,43	18,59 ± 1,02	16,68 - 20,74	23,69 ± 7,97	23,62 ± 1,42	20,81 - 26,54
RWSB18/GFP	LB	14,38 ± 13,34	14,56 ± 2,27	10,42 - 19,27	11,1 ± 13,36	11,07 ± 2,01	7,35 - 15,21
RWSB18/RFP	LB	22,67 ± 26,66	22,55 ± 2,64	17,26 - 27,58	20,89 ± 17,7	20,93 ± 3,2	14,77 - 27,4
ABS2408/GFP	LB	19,21 ± 10,27	19,33 ± 2,45	14,66 - 24,56	12,89 ± 10,91	12,95 ± 2,04	9,26 - 17,49
ABS2408/RFP	LB	24,57 ± 22,18	24,62 ± 2,82	19,3 - 30,37	13,53 ± 13,33	13,69 ± 2,65	8,8 - 18,93
NO50	LB	28,42 ± 9,36	28,5 ± 0,53	27,5 - 29,5	23,53 ± 10,4	23,5 ± 0,58	22,32 - 24,6
	S	18,53 ± 10,87	18,54 ± 0,57	17,48 - 19,68	20,84 ± 11,79	20,77 ± 0,71	19,35 - 22,13

Strains	Medium	% of constrained patches			% of unclassified patches		
NC103	LB	32,03 ± 13,94	32,03 ± 0,77	30,57 - 33,53	16,33 ± 9,51	16,21 ± 0,78	14,67 - 17,77
	S	40,3 ± 11,52	40,79 ± 1,23	38,48 - 43,18	8,51 ± 10,88	8,28 ± 0,89	6,55 - 10,03
	CH	19,48 ± 0,69	19,57 ± 4,43	11,56 - 28,28	16,1 ± 3,88	16, ± 5,29	6,13 - 26,71
	M9SE	32,81 ± 18,47	33,58 ± 2,97	27,55 - 39,31	14,43 ± 15,47	13,65 ± 2,56	8,54 - 18,77
2521	LB	31,09 ± 10,65	31,14 ± 0,77	29,56 - 32,58	15,41 ± 7,58	15,36 ± 0,72	13,98 - 16,68
	S	38,09 ± 12,77	38,38 ± 1,77	35,06 - 42,01	12,85 ± 14,	12,58 ± 1,53	9,48 - 15,34
	CH	39,77 ± 12,18	39,98 ± 2,06	35,96 - 44,04	13,56 ± 11,43	13,3 ± 2,08	9,27 - 17,31
	M9SE	50,9 ± 22,17	51,66 ± 2,01	47,84 - 55,87	4,99 ± 8,94	4,88 ± 1,04	2,93 - 6,97
RCL238	LB	35,97 ± 9,44	35,94 ± 1,23	33,61 - 38,38	18,27 ± 7,28	18,24 ± 1,13	16,11 - 20,5
2523	LB	44,92 ± 6,79	44,97 ± 1,55	42, - 47,84	12,76 ± 8,01	12,82 ± 1,13	10,59 - 14,97
RWSB18/GFP	LB	72,66 ± 19,73	72,53 ± 2,73	67,42 - 77,91	1,86 ± 5,15	1,83 ± 0,94	0,23 - 3,9
RWSB18/RFP	LB	55,55 ± 23,67	55,6 ± 3,76	48,38 - 62,7	0,89 ± 4,87	0,92 ± 0,83	0,0 - 2,59
ABS2408/GFP	LB	63,1 ± 14,02	62,96 ± 3,15	56,57 - 69,11	4,8 ± 12,7	4,77 ± 1,37	2,06 - 7,42
ABS2408/RFP	LB	56,33 ± 25,49	56,38 ± 3,25	49,96 - 62,62	5,57 ± 13,8	5,32 ± 1,59	2,13 - 8,37
NO50	LB	28,49 ± 9,63	28,58 ± 0,51	27,57 - 29,55	19,56 ± 11,85	19,42 ± 0,59	18,29 - 20,53
	S	52,03 ± 16,44	52,14 ± 0,8	50,48 - 53,69	8,59 ± 9,61	8,55 ± 0,52	7,55 - 9,58

M, measured mean (with standard deviation, SD); M*, bootstrap mean (with standard error, SE); Conf.interval., calculated confidence interval (95%) using the bootstrap method (see Supplementary methods).
Values are expressed in μm^{-2} (density), nm s^{-1} (speed) or %(distribution of MreB patches among different classes of movement)

Supplementary Table 5. Strains used in this study

Strain	Genotype	Construction or reference
E. coli		
TB28	*MG1655,* lacIZYA<>frt	[24]
NO50	*mreB :: mreB'-msfgfpsw-'mreB*	[25]
B. subtilis		
168	*trpC2*	laboratory collection
NC103	*trpC2, mreB::gfp-mreB, upp::*P_{Λ}*neo*	[17]
2521	*trpC2, mbl* Ω(*mbl-gfp cat*)	[26]
RCL238	*trpC2*, Δ*amyE*::(P_{xyl} *gfp-mreB spc*), *neo*-Δ*mreB*^{ml}	lab collection, sibling of 3723 [18]
2523	*trpC2*, Δ*amyE*::(P_{xyl} *gfp-mbl spc*), Δ*mbl*Ω(*pMUTIN4, erm*)	[27]
RWSB18	*trpC2, mbl* Ω(*mbl-mrfpruby cat*), *amyE*::(P_{xyl} *gfp-mreB spc*)	[18]
ABS2408	*trpC2, amyE*::(P_{xyl} *mrfpruby-mreB spc*), *mbl* Ω(*mbl-gfp cat*)	This study (2521 -> RWSB5)
RWSB5	*trpC2, amyE*::(P_{xyl} *mrfpruby-mreB spc*)	[18]
3725	*trpC2, neo*-Δ*mreB*^{ml}	[28]
RCL78	*trpC2*, Δ*mbl*::*cat*	This study (PCR -> 168)
ABS1324	*trpC2, mreBH* Ω(*mreBH-spa erm*)	This study (pAC616 -> 168)
CCBS121	*trpC2, mreB::gfp-mreB, upp::*P_{Λ}*neo, mreBH* Ω(*mreBH-spa erm*)	This study (ABS1324 -> NC103)
CCBS125	*trpC2, mbl* Ω(*mbl-gfp cat*), *mreBH* Ω(*mreBH-spa erm*)	This study (ABS1324 -> 2521)
CCBS126	*trpC2, neo*-Δ*mreB*^{ml}, *mreBH* Ω(*mreBH-spa erm*)	This study (ABS1324 -> 3725)
CCBS127	*trpC2*, Δ*mbl*::*cat, mreBH* Ω(*mreBH-spa erm*)	This study (ABS1324 -> RCL78)
NC91	*trpC2, amyE*::($P_{mreB123}$ *luc, cat*), p(*mcComS kan*)	[17]
NC94	*trpC2, amyE*::(P_{mbl12} *luc, cat*), p(*mcComS kan*)	[17]
NC96	*trpC2, amyE*::(P_{mreBH} *luc, cat*), p(*mcComS kan*)	[17]
NC84	*trpC2, amyE*::($P_{mreB123}$ *luc, cat*)	This study (NC91 -> 168)
NC87	*trpC2, amyE*::(P_{mbl12} *luc, cat*)	This study (NC94 -> 168)
NC89	*trpC2, amyE*::(P_{mreBH} *luc, cat*)	This study (NC96 -> 168)

^{ml} stands for marker-less deletion

Supplementary Table 6. Plasmids used in this study

Plasmid name	Description	Construction or reference
pMUTINspa	Dedicated to Campbell-like insert, at its natural locus in the *B. subtilis* genome, a gene fused in 3' to a SPA tag sequence, and to place downstream gene(s) under the IPTG-dependent P_{spac} promoter	[16]
pSweet	Integrative vector for *B. subtilis* carrying a chloramphenicol resistance cassette (cat)	[14]
pAC616	pMUTINspa derivative with a cloned 3' end fragment of *mreBH*	This study (AC933-938 -> pMUTINspa)

Supplementary Table 7. Primers used in this study

Primer name	sequence (5' to 3')	Purpose
RCL004	CTTCTTCAACTAACGGGGCAGGTTAGTGAC	*mbl* deletion (*cat* cassette)
RCL005	AATTCTCATGTTTGACAGCTTATCATCGGC	"
RCL012	GCCGCGGCCGCAATCGTAATCACCTGGCA	*mbl* deletion (upstream fragment)
RCL023	GTCACTAACCTGCCCCGTTAGTTGAAGAAGCCGCCAGCACTTTGCCGCTG	"
RCL025	GCCGATGATAAGCTGTCAAACATGAGAATTTACCGGGTCCTCGTTGCTAG	*mbl* deletion (downstream fragment)
RCL027	GCTGTATATAATCCTTTTAACAAATTTCCC	"
AC-938	AAGAAGCTTAATTTGCTGATCGGCGAACGCACAG	PCR fragment of *mreBH*, *Hind*III restriction site
AC-933	CCACCATGGTTTAATTGCCTTTTGCAGCTTATCAATC	PCR fragment of *mreBH*, *Nco*I restriction site

Supplementary Table 8. Calculated Full turn (FT) of MreB patches around the cell perimeter over one cell cycle

Strain	Medium	Cell parameters			Directed patches			FT
		τ	L	D	N_d	ρ_d	ν	
B. subtilis MreB	LB	28.3	4.7	0.88	4.86	0.46	59.6	**178**
	S	56.8	2.48	0.83	1.83	0.43	34.34	**82**
B. subtilis Mbl	LB	30.5	5.05	0.84	5.55	0.50	51.76	**199**
	S	60.4	2.51	0.82	2.13	0.49	29.91	**90**
E. coli MreB	LB	20.2	3.84	1.12	5.55	0.58	30.3	**58**
	S	54.7	2.97	0.72	2.23	0.44	26.91	**87**
		min	µm	µm		μm^{-2}	$nm\ s^{-1}$	

Supplementary Table 9. Estimated width of PG bands inserted per MreB patch

Strain	Medium	$L - D\ /\ FT$ (nm)	$ln(2)/(\nu\rho_d\tau)$ (nm)
B. subtilis MreB	LB	21.47	14.88
	S	20.05	13.90
B. subtilis Mbl	LB	21.15	14.66
	S	18.87	13.08
E. coli MreB	LB	45.42	31.49
	S	25.76	17.86

Supplementary Table 10. Estimated amount of PG inserted per MreB patch

Strain	Medium	**ω (nm)** $ln(2)/(\nu\rho_d\tau)$	**PG units**(*) (ω/4.5)	**PG corrected** (2 x PG unit)	**Triplets**
B. subtilis MreB	LB	14.9	3.31	6.61	2.2
	S	13.9	3.09	6.18	2.1
B. subtilis Mbl	LB	14.7	3.26	6.52	2.2
	S	13.1	2.91	5.81	1.9
				(1.5 x PG unit)	
E. coli MreB	LB	31.49	7.0	10.5	3.5
	S	17.86	3.97	5.95	2.0

(*) Estimated peptidoglycan unit length = 4.5 nm (estimated width of a stretched sugar strand plus a peptide cross-link).

Supplementary References

1 Ouzounov, N. *et al.* MreB Orientation Correlates with Cell Diameter in *Escherichia coli*. *Biophys.J.* **111**, 1035-1043, doi:10.1016/j.bpj.2016.07.017 (2016).

2 Vollmer, W. & Seligman, S. J. Architecture of peptidoglycan: more data and more models. *Trends Microbiol.* **18**, 59-66, doi:10.1016/j.tim.2009.12.004 (2010).

3 Vollmer, W. & Holtje, J. V. The architecture of the murein (peptidoglycan) in gram-negative bacteria: vertical scaffold or horizontal layer(s)? *J. Bacteriol.* **186**, 5978-5987, doi:10.1128/JB.186.18.5978-5987.2004 (2004).

4 Koch, A. L. Orientation of the peptidoglycan chains in the sacculus of *Escherichia coli*. *Res. Microbiol.* **149**, 689-701 (1998).

5 De Boer, W. R., Kruyssen, F. J. & Wouters, J. T. Cell wall turnover in batch and chemostat cultures of *Bacillus subtilis*. *J. Bacteriol.* **145**, 50-60 (1981).

6 Doyle, R. J., Chaloupka, J. & Vinter, V. Turnover of cell walls in microorganisms. *Microbiol. Rev.* **52**, 554-567 (1988).

7 Mobley, H. L., Koch, A. L., Doyle, R. J. & Streips, U. N. Insertion and fate of the cell wall in *Bacillus subtilis*. *J. Bacteriol.* **158**, 169-179 (1984).

8 Pooley, H. M. Layered distribution, according to age, within the cell wall of *Bacillus subtilis*. *J. Bacteriol.* **125**, 1139-1147 (1976).

9 Pooley, H. M. Turnover and spreading of old wall during surface growth of *Bacillus subtilis*. *J. Bacteriol.* **125**, 1127-1138 (1976).

10 Burman, L. G. & Park, J. T. Molecular model for elongation of the murein sacculus of *Escherichia coli*. *Proc. Natl. Acad. Sci. U S A* **81**, 1844-1848 (1984).

11 Holtje, J. V. Growth of the stress-bearing and shape-maintaining murein sacculus of *Escherichia coli*. *Microbiol. Mol. Biol. Rev.* **62**, 181-203 (1998).

12 Nguyen, L. T., Gumbart, J. C., Beeby, M. & Jensen, G. J. Coarse-grained simulations of bacterial cell wall growth reveal that local coordination alone can be sufficient to maintain rod shape. *Proc. Natl. Acad. Sci. U S A* **112**, E3689-3698, doi:10.1073/pnas.1504281112 (2015).

13 Schirner, K. & Errington, J. The cell wall regulator {sigma}I specifically suppresses the lethal phenotype of mbl mutants in *Bacillus subtilis*. *J. Bacteriol.* **191**, 1404-1413, doi:10.1128/JB.01497-08 (2009).

14 Bhavsar, A. P., Zhao, X. & Brown, E. D. Development and characterization of a xylose-dependent system for expression of cloned genes in *Bacillus subtilis*: conditional complementation of a teichoic acid mutant. *Appl. Env. Microbiol.* **67**, 403-410, doi:10.1128/AEM.67.1.403-410.2001 (2001).

15 Zeghouf, M. *et al.* Sequential Peptide Affinity (SPA) system for the identification of mammalian and bacterial protein complexes. *J. Proteome Res.* **3**, 463-468 (2004).

16 Lecointe, F. *et al.* Anticipating chromosomal replication fork arrest: SSB targets repair DNA helicases to active forks. *EMBO J.* **26**, 4239-4251, doi:10.1038/sj.emboj.7601848 (2007).

17 Mirouze, N., Ferret, C., Yao, Z., Chastanet, A. & Carballido-Lopez, R. MreB-Dependent Inhibition of Cell Elongation during the Escape from Competence in *Bacillus subtilis*. *PLoS Genet.* **11**, e1005299, doi:10.1371/journal.pgen.1005299 (2015).

18 Dominguez-Escobar, J. *et al.* Processive movement of MreB-associated cell wall biosynthetic complexes in bacteria. *Science* **333**, 225-228, doi:10.1126/science.1203466 (2011).

19 Vrljic, M., Nishimura, S. Y., Brasselet, S., Moerner, W. E. & McConnell, H. M. Translational diffusion of individual class II MHC membrane proteins in cells. *Biophys. J.* **83**, 2681-2692, doi:10.1016/S0006-3495(02)75277-6 (2002).
20 Schutz, G. J., Schindler, H. & Schmidt, T. Single-molecule microscopy on model membranes reveals anomalous diffusion. *Biophys. J.* **73**, 1073-1080, doi:10.1016/S0006-3495(97)78139-6 (1997).
21 Schindelin, J. *et al.* Fiji: an open-source platform for biological-image analysis. *Nat. Methods* **9**, 676-682, doi:10.1038/nmeth.2019 (2012).
22 Efron, B. & Tibshirani, R. Statistical data analysis in the computer age. *Science* **253**, 390-395, doi:10.1126/science.253.5018.390 (1991).
23 Efron, B. & Tibshirani, R. *An introduction to the bootstrap*. (Chapman & Hall, 1993).
24 Bernhardt, T. G. & de Boer, P. A. The *Escherichia coli* amidase AmiC is a periplasmic septal ring component exported via the twin-arginine transport pathway. *Mol. Microbiol.* **48**, 1171-1182 (2003).
25 Morgenstein, R. M. *et al.* RodZ links MreB to cell wall synthesis to mediate MreB rotation and robust morphogenesis. *Proc. Natl. Acad. Sci. U S A* **112**, 12510-12515, doi:10.1073/pnas.1509610112 (2015).
26 Jones, L. J., Carballido-Lopez, R. & Errington, J. Control of cell shape in bacteria: helical, actin-like filaments in *Bacillus subtilis*. *Cell* **104**, 913-922 (2001).
27 Carballido-Lopez, R. & Errington, J. The bacterial cytoskeleton: in vivo dynamics of the actin-like protein Mbl of *Bacillus subtilis*. *Dev. Cell* **4**, 19-28 (2003).
28 Formstone, A. & Errington, J. A magnesium-dependent *mreB* null mutant: implications for the role of *mreB* in *Bacillus subtilis*. *Mol. Microbiol.* **55**, 1646-1657, doi:10.1111/j.1365-2958.2005.04506.x (2005).
